# Intelligent User Interfaces and Their Evaluation: A Systematic Mapping Study

**DOI:** 10.3390/s22155830

**Published:** 2022-08-04

**Authors:** Saša Brdnik, Tjaša Heričko, Boštjan Šumak

**Affiliations:** Faculty of Electrical Engineering and Computer Science, University of Maribor, 2000 Maribor, Slovenia

**Keywords:** intelligent user interfaces, IUI, usability, user experience, evaluation

## Abstract

Intelligent user interfaces (IUI) are driven by the goal of improvement in human–computer interaction (HCI), mainly improving user interfaces’ user experience (UX) or usability with the help of artificial intelligence. The main goal of this study is to find, assess, and synthesize existing state-of-the-art work in the field of IUI with an additional focus on the evaluation of IUI. This study analyzed 211 studies published in the field between 2012 and 2022. Studies are most frequently tied to HCI and SE domains. Definitions of IUI were observed, showing that adaptation, representation, and intelligence are key characteristics associated with IUIs, whereas adaptation, reasoning, and representation are the most commonly used verbs in their description. Evaluation of IUI is mainly conducted with experiments and questionnaires, though usability and UX are not considered together in evaluations. Most evaluations (81% of studies) reported partial or complete improvement in usability or UX. A shortage of evaluation tools, methods, and metrics, tailored for IUI, is noticed. Most often, empirical data collection methods and data sources in IUI evaluation studies are experiment, prototype development, and questionnaire.

## 1. Introduction

Throughout the decades, the science fiction film industry has produced an infinite number of ideas and concepts of complex, artificial intelligence-driven (AI) user interfaces (UIs). Science fiction is compelling because it can show us what is possible when you are unconstrained by technology in finding ideas for novel, complex, AI-driven user interfaces. However, the power of science fiction is not in entertainment but in inspiring people with what is possible, what is ideal, and what is astonishing [1]. Especially for computer science and AI, science fiction movies have provided a vast number of ideas for developing innovative, intelligent user interface (IUI) solutions. For example, HAL 9000, which appeared in the 1968 movie 2001: A Space Odyssey, was a Heuristically programmed ALgorithmic computer able to control the Discovery One spacecraft’s systems and interact with the ship’s crew. In a scene where Dave returns to the ship after the incident caused by HAL with the crew, HAL said to Dave, *“Look Dave, I can see you’re really upset about this. I honestly think you ought to sit down calmly, take a stress pill, and think things over. I know I’ve made some very poor decisions recently, but I can give you my complete assurance that my work will be back to normal. I’ve still got the greatest enthusiasm and confidence in the mission. And I want to help you.”* [2]. In this science fiction movie, HAL was able to recognize Dave, analyze his speech, and recognize his emotions. HAL’s capabilities, introduced in the movie, were based on ideas of various respected scientists and the most influential researchers in fields such as computer science, speech recognition, language understanding, computer vision, emotion recognition from facial expression, AI, etc. [3]. Among others, HAL has probably inspired and influenced research in the decades that followed and brought today’s machines capabilities that are even far more sophisticated than those presented in the movie. Because of significant advancements in sensing and AI technologies, several decades later, HAL’s capabilities are no longer science fiction [4].

In this article, we present an overview of the IUI field in the last decade in the form of a systematic mapping study. Additional insight is provided into current trends in the evaluation of intelligent interfaces. In existing IUI-related research, systematic literature reviews and systematic mapping studies have investigated, for example, smart, context-sensitive, and multimodal user interfaces [5], adaptive user interfaces [5,6,7,8], intelligent human–computer interaction [9], and adaptive and adaptable user interfaces [8]. Because the focus of our work is on IUI solutions, our study can be considered close to the work performed by Volkel et al. [10]. However, the meta-analysis conducted by [10] was restricted to research published in the ACM International Intelligent User Interface conference proceedings. To our best knowledge, this study is one of the first to provide a systematic review and mapping of existing research published in the last decade in the field of IUI, focusing on IUI usability and user experience (UX) evaluation methods. This article can enable both IUI and UX researchers to gain broader and deeper insight into IUIs’ UX and usability evaluation research in the last decade by facilitating researchers to understand novel methods and techniques used in IUI evaluation and find future research directions. The main contributions in this study are threefold: (i) Presenting an overview of the observed IUI filed, (ii) Contributing to the debate what solutions are deemed intelligent in IUI field with the analysis of definitions in use and solutions proposed as intelligent, and (iii) recognizing research gaps in IUI evaluation.

The remainder of this article is organized as follows. Section 2 provides a short overview of the background and related work, and Section 3 details the structure of the systematic mapping study methodology. Results are presented in Section 4 with an additional discussion provided in Section 5. We conclude the article with Section 6, where the main conclusions are summarized and future directions are suggested.

## 2. Background and Related Work

### 2.1. Intelligent User Interfaces

The idea of introducing intelligence to human–computer interaction (HCI) and user interface sprouted decades ago in the form of intelligent computer-assisted instructions, which later gained a wider following and application as IUIs [11]. The field connects different disciplines, mainly AI, software engineering (SE), and HCI, with AI contributing simulation and prediction techniques for communication improvement, and HCI contributing insight into users, their behavior, and needs.

Though a universal understanding of IUI definition would be expected with observed growth in the number of studies published in the last decade and a long history of the field, recent studies [10] instead show a mixed understanding of the term and its boundaries, resulting in a non-uniformed use. IUI are human–machine interfaces that aim to improve the efficiency, effectiveness, and naturalness of HCI by representing, reasoning, and acting on models of the user, domain, task, discourse, and media [11]. In the context of adaptation, there are some similar types of user interfaces. *Adapted user interfaces* [12] are user interfaces adapted to the end-user at design time, with no adaptation changes occurring in run time. *Adaptable user interfaces* [12] allow users to provoke changes (i.e., trigger manual adaptations) to the characteristics or functionalities of the user interface. In contrast, *adaptive user interfaces* (AUI) change their characteristics dynamically at run time with changes being triggered by the user’s behavior [12]. They monitor user behavior patterns and create and maintain user profiles, using them as the base for in-time adaptation [13]. The adaptations can be triggered for individual users or groups of users, adapting their layout and components according to the user context [14]. The term intelligent user interface is often used along with various adapt* terms, as reported by a meta-study conducted by Volkel et al. [10], where authors confirmed that the studies might call an entity both “intelligent” and “adaptive”. The concurrence can even be observed in use of the term *adaptive intelligent user interfaces* [15]. Though this term is used infrequently, it describes user interfaces with intelligent adaptive mechanisms capable of monitoring the user behavior and adapting the user interface accordingly, outside of the predefined rules. Many intelligent interfaces can be described as adaptive interfaces, though not all adaptive interfaces are intelligent. IUIs can be associated with *intelligent systems*, i.e., systems that give appropriate problem-solving responses to problem inputs, even if such inputs are new and unexpected. Therefore, their behavior can be described as “novel” or “creative” [16]. Intelligent systems cannot be labeled as IUI even if they utilize a user interface if they are only intelligent from a machine standpoint but not from a user or HCI standpoint [17].

#### Evaluation of Usability and User Experience

In the existing literature, various variations of usability and user experience (UX) definitions can be found. In this study, we understand the concept of usability as defined by the ISO [18] as an *“extent to which a system, product or service can be used by specified users to achieve specified goals with effectiveness, efficiency, and satisfaction in a specified context of use”*. Usability can be considered as a part of UX, which is defined by ISO [18] as *“user’s perceptions (including the users’ emotions, beliefs, preferences, perceptions, comfort, behaviors, and accomplishments that occur before, during and after use) and responses that result from the use and/or anticipated use of a system, product or service”*. For evaluated factors of usability, components defined by ISO 9241-400 [19] are the most widely used, including learnability, appropriateness, recognizability, operability, user error protection, user interface aesthetics, and accessibility. Furthermore, the standard states that usability criteria can be used to assess aspects of UX [18]. Factors proposed for evaluation in UX still vary in practice, though it is observed that they are more connected to affect, interpretation, and meaning [20]. Factors observed in studies evaluating UX [21] include appearance, perceptions, performance, availability, and overall satisfaction. Additionally, cognitive load [22] and efficacy [23] have also been observed as factors in intelligent user interface evaluation.

Evaluating IUIs has been a point of interest in the field for decades. The issues caused by introducing machine learning (ML) and adaptations to user interfaces are violations of good usability principles (e.g., giving users control over the system, making the system predictable and transparent), trust, and privacy [24]. Observed usability issues can outweigh the advantages introduced with intelligence and adaptations. Evaluating the adaptability of interfaces, Gajos [25] reported the greatest user satisfaction and performance improvement by increasing the accuracy over the predictability in user interfaces.

### 2.2. Artificial Intelligence Methods for IUIs

The development of IUIs greatly benefits from a number of AI fields’ methodologies, strategies, and concepts. AI methods have been introduced to the IUI field to create a more natural and efficient HCI. Based on how AI is included in IUIs, we recognize [26] intelligent assistants, intelligent tutoring systems, intelligent help and support systems, decision support systems, cooperative intelligent agents, and dialogue assistants. Mainly, AI is included in IUIs in the form of intelligent agents, algorithms, tools, and assistants [10]. ML has become one of the most utilized approaches in the AI field for developing useful software for computer vision, speech recognition, natural language processing, robot control, and other applications [27]. As it is learning from past data and offers an automatic improvement over time, bypassing the need for manually programming inputs and corresponding outputs, it offers new ways of newer, faster, and more accurate adaptation, recommendation, help, detection, illustration, and utilization in user interfaces.

Based on the learning style, ML algorithms and methods can be categorized into [28]: *(1) supervised learning algorithms*, which are ML techniques that can be applied according to what was previously learned to obtain new data using labeled data and to predict future events or labels (e.g., Classification, Support Vector Machine, Discriminant Analysis, Naïve Bayes, Neural Network, Extreme Machine Learning, Relevance Vector Machine, Gaussian Processes, Combined Algorithms, etc.), *(2) unsupervised learning algorithms* are algorithms with no supervisor to guide or correct, often used when unlabeled or unclassified information is present to train the system (e.g., Clustering, Hierarchical ML, Unsupervised Gaussian Mixture, Hidden Markov Model, K-means, Fuzzy C-means, Neural Networks, etc.), *(3) semi-supervised learning algorithms*, which are between the category of supervised and unsupervised learning that use both unlabeled and labeled data for training purposes (e.g., algorithms applying self-training techniques, graph-based semi-supervised learning algorithms, such as Graph Neural Networks, Graph Convolutional Networks, etc.), and *(4) reinforcement learning algorithms* that are types of learning methods that give rewards or punishment on the basis of the work performed by the system (e.g., model-based reinforcement learning, model-free reinforcement learning, etc.).

### 2.3. Related Work

In the last decade, several secondary studies connected to the field of IUI have been conducted. Differentiating in scope, some focused on the application of IUI’s in a particular field [6], whereas others [29] provided a wider interdisciplinary overview of intelligent systems. Although some parallels can be observed with existing work, the lack of focus on evaluation can be observed in the current works, whereas further characteristics make our study the first of its kind in the field.

Firstly, our study differentiates with the keywords used for the literature search. Previous studies included various other types of user interfaces in their review, such as smart, context-sensitive, and multi-modal user interfaces [5] or adaptive user interfaces [5,6,7,8]. Additionally, ref. [9] included intelligent interaction, whereas [29] expanded the review to transparency, explainability, and context interpretation. One of the secondary studies [8] focused only on adaptive and adaptable user interfaces. The meta-analysis of conference articles from the ACM International Intelligent User interface conference [10] provided a similar review in the aspect of the content with the focus on the keyword “intelligent”. However, the restriction to one, albeit primary, conference publication space does not necessarily give a clear picture of the whole field. Furthermore, none of the existing secondary studies focused solely on providing an overview of the IUI and their evaluation. A complete overview of related secondary studies is presented in Table 1.

A meta-analysis [10] of articles published in the ACM International Conference on Intelligent User Interfaces in the last 25 years, focused on intelligent entities in IUI articles, was conducted in 2020. The most commonly described intelligent entities remain interface, system, agent, assistant, tutoring system, and algorithm. One of the recognized challenges highlights the expanding need for evaluation and standards in the field. The co-descriptors most often used with the term are intelligent, autonomous, adaptive, interactive, dynamic, natural, and context-aware.

A systematic mapping study [6] was conducted in 2017, focused on adopting IUI technologies with Ambient Assisting Living technologies. Articles connected to adaptive and intelligent user interfaces were analyzed. Applications, graphics, and visual software were recognized as the most commonly presented software solutions with IUI. The evaluation of the IUI was, however, not analyzed. The presentation of the prototype was observed as the most commonly used validation method. In [5], the preliminary results of a systematic literature review were presented, with a focus on the design trends of IUIs and the means of their development. The rising number of IUI applications in the health domain was highlighted, and the context, dialogue, and user models were presented as most cited in all observed solutions. Five categories of the user interface were recognized: adaptive user interface, context-sensitive user interface, intelligent user interface, multi-modal user interface, and smart user interface, with the former being the most used expressions.

Systematic literature review [7] analyzed universal usability through plasticity, focusing on adaptive and adaptable interfaces, where the trend toward adaptive user interfaces was recognized. The adaptation toward individual users’ needs was observed, especially the mental workload and stress levels. To observe the trade-off that adaptive context-aware interfaces present for usability, a performance evaluation of the plasticity of the user interface was conducted in the second part of the study. The research gap in determining the usefulness and effectiveness of IUIs were recognized, and the need for the optimization of the usability trade-offs was indicated. The research gap in comparing the usefulness and effectiveness of IUIs compared to AUIs is also noted.

In one of the broadest interdisciplinary literature analyses, Ref. [29] analyzed more than twelve thousand articles from various research areas, such as HCI, psychology, and artificial intelligence, with a focus on the trends in explainable systems and explainable AI. Research clusters connecting these fields are recognized, mainly causality and psychology of explanations; algorithmic fairness, transparency, and interpretable machine learning; intelligent and ambient systems; interactions, software learnability, and ambient and intelligent systems. The trend of migrating from Bayesian Networks clusters to interpretable ML for explanations and the trend of focusing on gaining individual trust to moving toward institutional trust are described. Explanations of cognitive psychology, interaction design, and software learnability in combination with empirical evaluations are suggested as means of improving usability.

User-centered evaluations of adaptive and adaptable systems were analyzed in [8] in 2008, with usability, perceived usefulness, and appropriateness of adaptation being the three most commonly assessed variables in observed primary studies. At the time, questionnaires were the most popular method for evaluating adaptive and adaptable interfaces, followed by interviews and data log analysis. The evaluation mainly focused on the personalized systems.

A similar systematic mapping study researching usability and UX evaluation was conducted with the focus on natural user interfaces [30]. It was discovered that the observed technologies evaluated only one aspect of natural user interfaces; usability or UX but did not consider them together. Most of the evaluations were quantitative, and the most commonly evaluated aspects of usability and UX were user satisfaction, effectiveness, efficiency, and performance. In a recent and related systematic mapping study [9], the focus was widely placed on identifying and analyzing the state-of-the-art AI methods and algorithms and sensors technology in existing human–computer intelligent interaction (HCII) research to explore trends in HCII research. The primary focus in HCII studies has been intelligent recognition of emotion, gestures, and facial expressions using sensors technology, such as the camera, EEG, Kinect, wearable sensors, eye tracker, and gyroscope. A further research gap in evaluating the usability and UX of similar studies was indicated.

## 3. Materials and Methods

**Figure 1:**
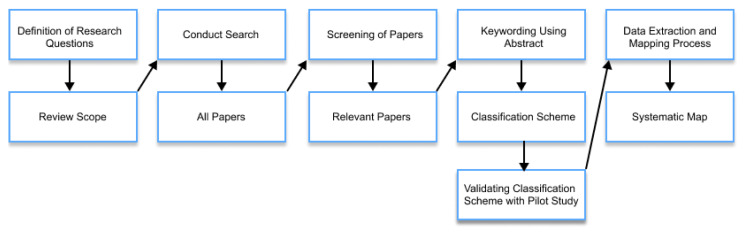
The systematic mapping study process adapted from Petersen et al. [31].

This study was conducted with the goal of structuring and categorizing studies on IUIs and the evaluation of said interfaces. The protocol for conducting a systematic mapping study, written by Petersen et al. [31], was followed. The process of obtaining and analyzing primary studies is presented in Figure 1. The deviation from the protocol should be noted in the data extraction step, as it was based on the review of the entire articles, not just the abstract and keyword screening. The protocol was also extended with the iterative improvement of the classification scheme during the pilot study, conducted on 40 articles.

The research process in this study was divided into five phases. Firstly, the research questions presented in Table 2 were defined. Secondly, the scope of the study was reviewed with the preliminary research in the selected databases, with the number of papers retrieved from selected digital libraries presented in Table 3. Next, inclusion and exclusion criteria, presented in Table 4 were defined and used. Number of studies, observed in each step is presented in Table 5. Based on the review of the related work and our research questions, the classification scheme was defined. Next, the screening of articles based on the abstract was conducted, and relevant articles were extracted for further reading. The classification scheme was revised and extended at this point, with the final version presented in Table 6. In the next step, the data extraction was executed based on reading the entire article. Lastly, multiple visualizations in the form of systematic maps were created as presented in the results in Section 4.

### 3.1. Definition of Research Questions

The study’s main goal is to find, assess, and synthesize existing state-of-the-art work in the field of IUI with an additional focus on evaluating IUI. The main goal of this research is threefold; (1) to perform a systematic mapping study of the IUI area by carefully reviewing important state-of-the-art scientific publications, (2) to recognize trends and conduct a demographic analysis of the IUI research field, and (3) to provide an overview of the current state and trends of IUI evaluation. We developed three key research questions based on the research goal: RQ1, RQ2, and RQ3. Further sub-questions were presented for better organization of the in-depth overview. Research questions and their corresponding subquestions are given in Table 2.

### 3.2. Conducting Search and Screening

Based on the determined research questions and overview of related work, the suitable keywords for discovering all published works with IUI themes were defined. Wide keywords were utilized to offer a thorough review of the study field because we wanted to present a comprehensive picture of the research topic. The following query was used for discovering primary studies:

*“intelligent user interfaces” OR “intelligent user interface” OR “IUI”*

In the process of the data source selection, the relevance and size of digital libraries were considered as well as protocols from related studies. Selected databases are the ACM Digital Library, IEEEXplore, ScienceDirect, Scopus, and Web of Science. The search was conducted on 20 January 2022. Altogether, 9849 possibly relevant articles were acquired. The highest number of research articles that may be relevant was found in the Web of Science digital library.

The scope of possibly relevant research was reduced in the next step based on predefined inclusion and exclusion criteria, which are presented in Table 4. After introducing the inclusion criteria I1–I4, the number of relevant studies was reduced to 1036, as presented in Table 5. Multiple articles have appeared in different repositories. In these cases, they were considered only once in the review and were associated with the first repository appearance, according to the performed search order (IEEEXplore, ACM Digital Library, Scopus, Web of Science, ScienceDirect). Removing the duplicates reduced the number to 607, and screening the abstracts reduced it to 376. After this step, the classification was conducted, and some additional studies were removed, mostly due to the article length or focus outside of the IUI field. The final scope of primary studies included in our review is 211 articles.

### 3.3. Classification and Data Extraction

The data extraction strategy was designed to provide answers to research questions presented in the previous section (Table 2). To ensure consistency in the classification process, the rules about coding data regarding study characteristics and the results were specified in advance. The classification scheme with specific extraction variables is outlined in Table 6.

We classified chosen studies by *research type* as suggested by [32] as validation research, evaluation research, solution proposal, philosophical article, opinions article, and experience article. We have further expanded this classification with a category literature review, which allowed us to include the secondary studies from our sample. The research type should not be confused with many used research methods, especially in the case of evaluation. Including limited evaluation methods (usually, as a secondary or tertiary research method used in the article) does not necessarily mean that the study is considered an evaluation article. If the scope of the evaluation in the view of the entire study was limited (e.g., work performed in the lab [31]), it should not be considered an evaluation study. The main difference between evaluation and validation research is that the first describes methods not yet implemented in practice being investigated, whereas the second investigates techniques-in-practice, mainly outside of the laboratory setting [32].

Secondly, we noted the *publication type* as a journal article or conference article, with proceedings articles being counted as conference articles. As noted in the previous section, IUI is a multidisciplinary field with an emphasis on two core disciplines—ACI and AI. Patrick [33] describes fields connected to IUI even further, noting that psychology, ergonomics, human factors, cognitive science, and social sciences also influence the field. This study focuses on the two core disciplines with the *standpoint* variable, where we noted if the research was conducted from a SE/AI point of view, from HCI, or if both domains were represented in the article. With the *methodology* variable, we documented the research methods used in the article. A variable *primary research strategy* was used to describe the main research approach in the observed studies. Only the prevalent one was extracted if multiple approaches were used in the study. In variable *data acquisition methods*, all prevalent methods used for acquisition of data as input for research were extracted. If the study proposed or analyzed any kind of software solution, the data were extracted as *Software type*. Further on, the engineering phase of the proposed solution was noted (if applicable) in the *Engineering phase*. Classification variable *domain* was used to describe the article’s primary application domain. Values for applicable domains were primarily gathered from related work [5,6,9] and expanded in the pilot study. If the study used any IUI definitions, they were extracted to EC12 verbatim, together with the source used in the definition, if the source was mentioned. If described solutions were part of any contemporary technological system, this was noted in EC13.

The intelligent entity was extracted to the variable *intelligent entity* as identified by the study’s authors. Only the primary intelligent entity was identified for each study. In cases where the system was noted as a primary intelligent entity, additional checkup was performed to assure the existence of intelligence in user interfaces, as well as in the system. The used artificial intelligence methods and algorithms were extracted in EC14 and classified. In cases where machine learning was utilized, the classification of algorithm or approach was also noted. ML techniques were later classified according to utilized learning style [34,35] in (i) supervised learning algorithms, (ii) unsupervised learning algorithms, (iii) semi-Supervised Learning, and (iv) reinforcement learning algorithms.

### 3.4. Analysis of IUI Definitions

Analysis of extracted IUI definitions was conducted based on the simplified protocol used in [36], where one hundred definitions of Industry 4.0 were reviewed. First, we extracted definitions and their sources from primary studies. If multiple instances of definitions were found, they were extracted separately. Then, a separate classification framework was used to determine the scope and defining elements in IUI definitions. Observing scope, we classified them as (i) field and domain definitions (positioning the IUI in a larger research area), (ii) user interface definitions (i.e., definitions of characteristics of user interfaces that are intelligent), (iii) ad-hoc definitions, which focused on short descriptions of IUI properties that were important for a particular study, and (iv) technology definitions, which focused on the technical aspect of IUIs. Further, the distinctive characteristics were extracted, followed by actions performed by IUI and any mentions of technologies crucial for IUI (i.e., AI and ML). Lastly, details on adaptation were extracted, mainly what is adapted and to what triggers the interface adaptation. The classification scheme is presented in Table 7.

## 4. Results

In this section, the results obtained from the analysis of the 211 primary studies are presented. The overview of all included studies and their classification (including essential classification variables only) is available as Supplementary Material.

### 4.1. Trend and Demographics in the IUI Field

**Figure 2:**
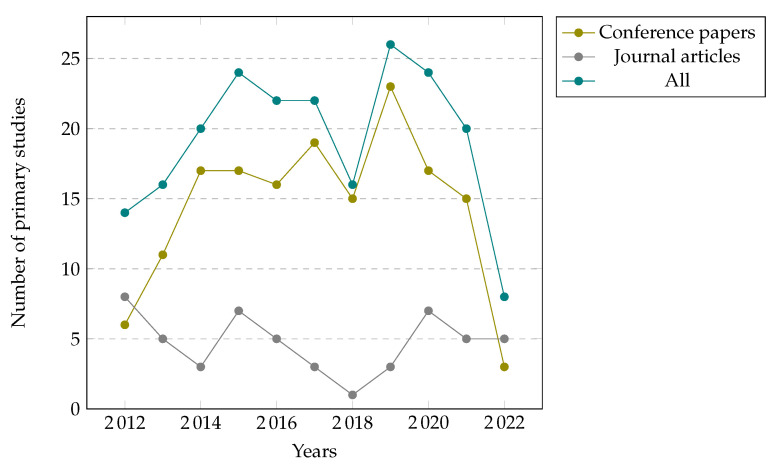
Type of primary studies by year (N = 211).

**Figure 3:**
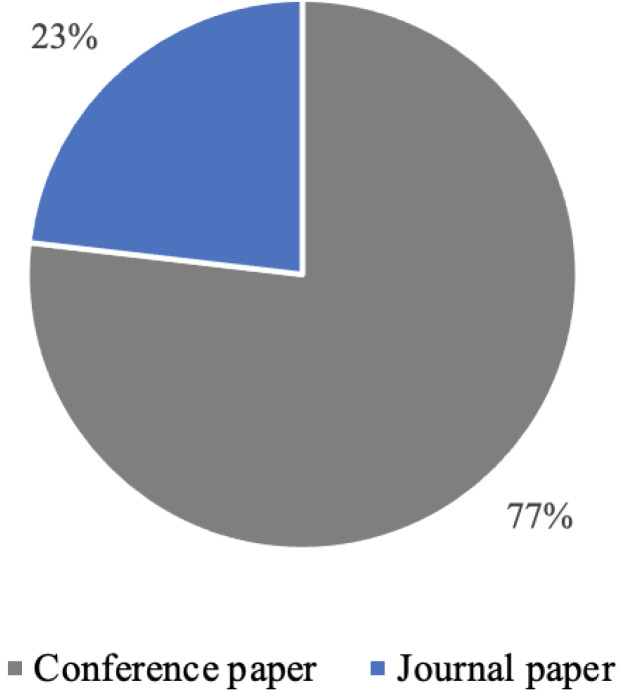
Primary studies by type (N = 211).

As visualized in Figure 2, we can observe an increasing trend in the number of conference and journal articles published up to 2020. The data for the year 2022 is incomplete due to the primary studies gathering being concluded in January 2022. Some minor decreases in the number of published studies can be observed in 2020 and 2021. However, the number of journal articles published in January of 2022 in the IUI field reached the overall number of articles published last year, suggesting increased interest in the field. The majority (76%) of the articles have been published as conference articles, as illustrated in Figure 3.

Table 8 provides an overview of publications and conferences where observed articles on IUI were published. Most represented journals in the IUI field are ACM Transactions on Interactive Intelligent Systems (nine articles), interacting with computers (three articles), and IEEE Access (two articles). All of the journals mentioned are peer-reviewed. The leading conference for publications from the IUI field is the International Conference on Intelligent User Interfaces (44 articles), followed by proceeding articles published in Lecture Notes in Computer Science (19 articles) and CEUR Workshop proceedings (10 articles).

**Figure 4:**
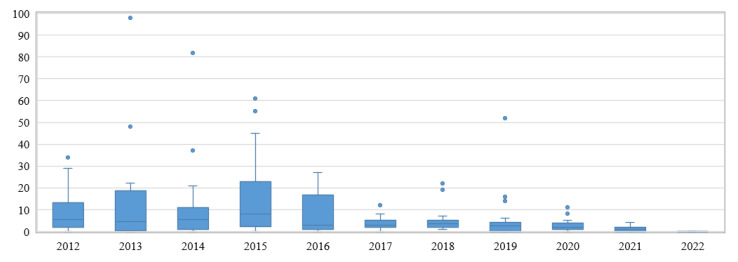
Number of citations by year.

The top ten most cited articles from the observed studies are presented in Table 9. Five of them have been published at the ACM’s International Conference on Intelligent User Interfaces, whereas the most cited article (S183) was published in the journal IEEE Transactions on Multimedia. To combat the age effect in article citations, average number of yearly citations is also noted. Generally, the number of citations increases in the first years after publication reaches a peak, and then the articles are less cited as time passes. Further insight into the citation trends through the years in the IUI field is provided in Figure 4, where the trend of a higher number of accumulated citations in older studies is visible. As suggested by Bornmann [37], recent studies published in the last two years were separated in the bibliometric analysis in order to avoid spurious or misleading findings. The average number of citations in the observed studies from non-recent years (2012–2019) is 9.1 (N = 160, SD = 14.1). In observed non-recent articles, journal articles had, on average, a higher number of citations (M = 9.4, N = 52, SD = 17.1) than conference articles (M = 8.1, N = 124, SD = 13.2) and magazine articles (four citations).

To gain a better picture of who is contributing to the field and scientifically promoting IUI subjects, we looked at the institutions of the initial authors and their locations. Most primary institutions are located in the USA (56 articles), Germany (25 articles), the United Kingdom (13 articles), China (9 articles), Japan (8 articles), France (7 articles), and Ireland (6 articles). The majority of researchers in the IUI field are active in Europe (42%)– and—North America (28%), followed by Asia (12%). The overview of top countries regarding the contributions to the IUI field is presented in Table 10.

To address the ongoing issue of the non-uniformly used definitions of IUI, we extracted the definitions used in primary articles. Overall, 38 instances of IUI definitions were extracted, with nine articles presenting two definitions. Most representative by the number of times used was—Maybury’s [11] original definition, used by three articles (S34, S56, and S138); *“Intelligent user interfaces (IUIs) are human-machine interfaces that aim to improve the efficiency, effectiveness, and naturalness of human-machine interaction by representing, reasoning, and acting on models of the user, domain, task, discourse, and media (e.g., graphics, natural language, gesture).”* Some studies used ad-hoc, generalized, or dictionary definitions. The majority of the used definitions included the term adapt* (21 definitions), thirty-one mentioned user*, six used some variation of the term model*, and a further six used a variation of the term understand*.

**Figure 5:**
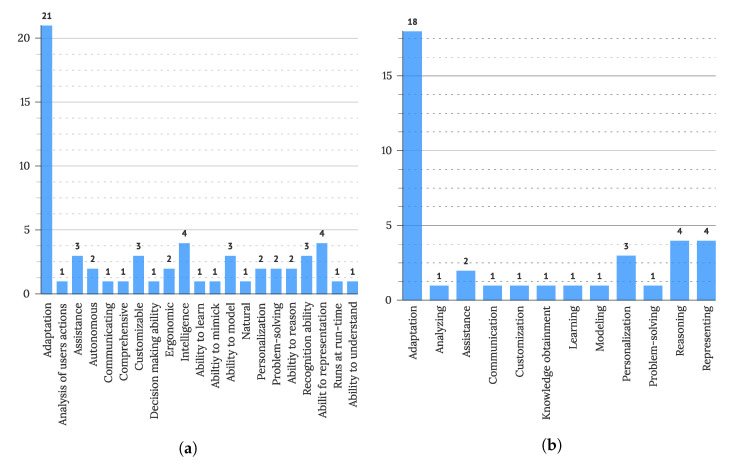
(**a**) Characteristics and descriptors of IUI and (**b**) Actions of IUI as extracted from definitions.

Detailed definition analysis, described in Section 3.4, was conducted on 38 extracted definitions. Extracted definitions and classification schema-based categorization of each definition are available in Appendix A. Observing scope, most of the definitions focused on the user interface (21 studies), many were ad-hoc (12 studies), used only to communicate the main idea of IUIs, a further four focused on the technological aspect of the technology, and two focused on—placing the field in the wider research area. Defining characteristics for IUI are presented in Figure 5, with the most distinct characteristic recognized being adaptation (mentioned in 53% of observed definitions). Other defining characteristics were more dispersed through definitions, though representing (4 definitions), intelligence (4 definitions), recognition (3 definitions), customization (3 definitions), and assistance (3 definitions) were also mentioned multiple times. From the technological point of view, one-fourth of observed definitions (10 definitions) described the connection between IUIs and AI– and a further seven observed connections to intelligence. Two definitions (S35, S153) further recognized the connections with natural language processing (i.e., a branch of ML) as a narrower possible definition of IUIs. Interestingly, one ad-hoc definition (S66) connected IUIs with ML, not the general AI area as considered in other definitions. Analyzing actions that definitions associate with IUIs, the connection with adaptation was again the strongest, recognized in almost half of the definitions (48% or 18 definitions), followed by reasoning (4 definitions), representing (4 definitions), personalization (3 definitions), and assistance (2 definitions). Learning, analyzing, communication, customization, modeling, and problem-solving were also each mentioned once, as displayed in Figure 5.

As adaptation was the central theme observed in definitions, further observations of what IUIs adapt to and what is being adapted were conducted, showing that most definitions recognize adapting to users (N = 13) and users’ needs (N = 5) as central goals of IUI adaptations. Other drivers of adaptation were context (N = 4), situation (N = 3), task (N = 3), user’s intent (N = 3), goals (N = 2), human behavior (N = 2), media (N = 2), discourse (N = 2), domain (N = 2), and device (N = 2). Attention, behavior patterns, scenario, and environment were also mentioned in one definition. In terms of adapted elements or characteristics, adaptations of user interface or UI elements (11 definitions) and interaction (4 definitions) were most often mentioned, followed by behavior (2 definitions). Adaptations of response, system, and response were also each mentioned once.

### 4.2. Research Space

**Figure 6:**
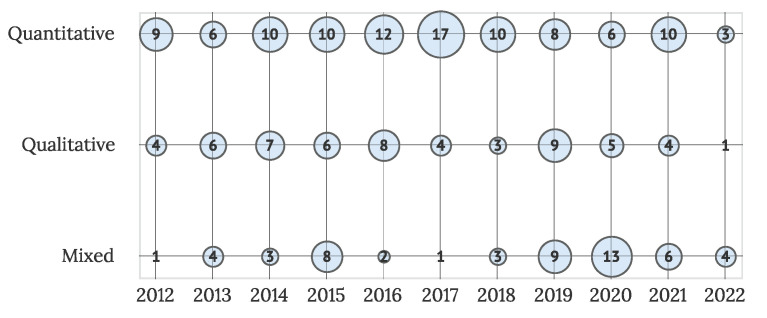
Number of studies by research type and year (N = 211).

To provide some insight into the research space in the IUI field, the overview of the methods in selected primary studies was conducted. Most of the studies (101 articles) are primarily quantitative, followed by qualitative (57 articles) and mixed studies (54 articles). A detailed overview of research type by year is presented in Figure 6. The trend of quantitative studies is visible in all observed years, with some increases in the number of published mixed research also visible in the last few years.

**Figure 7:**
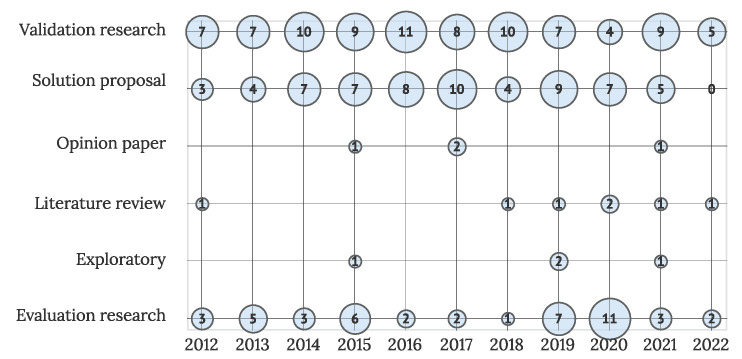
Studies by research methodology and year (N = 211).

The research type classification of primary studies is presented in Figure 7. The majority of the primary studies (87 articles) are validation articles, followed by solution proposals (64 articles)– and evaluation research (45 articles). Four exploratory and opinion articles were included, along with seven literature reviews. The trend of publishing validated solutions is visible in the field, accompanied by the slowly increasing number of evaluation studies in recent years. The number of solution proposals has remained relatively constant over the years.

**Figure 8:**
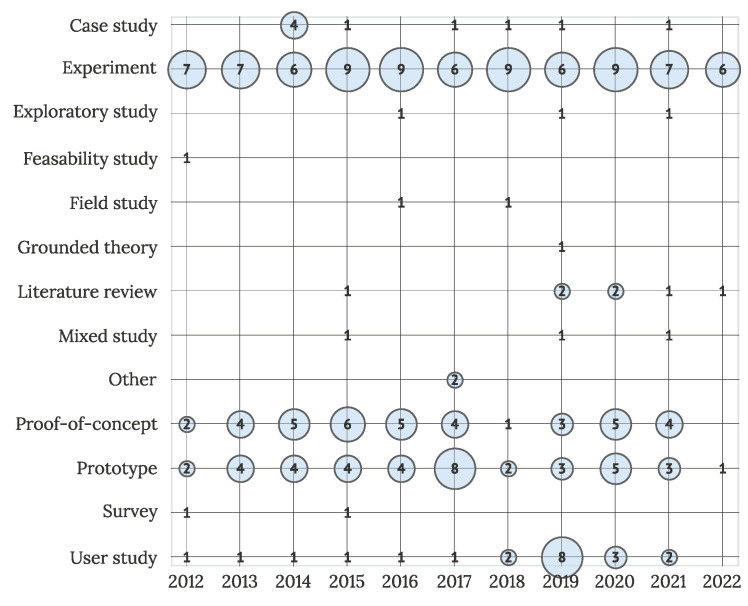
The primary research strategy used in research (N = 211).

To provide some more insight into the research scope, the primary research strategies used in the observed studies are visualized in Figure 8. Most commonly, researchers used experiments (N = 81), prototypes (N = 40), proof-of-concept (N = 39), and user studies (N = 21) as primary research strategies.

**Figure 9:**
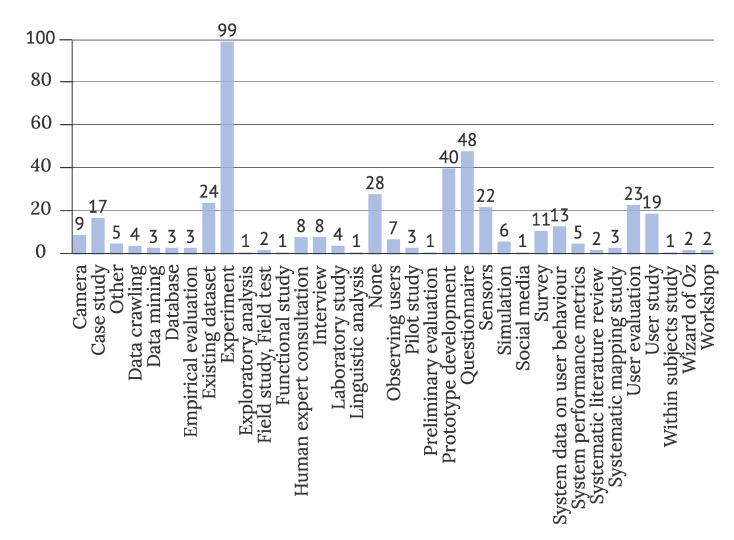
Data sources used in studies (N = 438).

Visualization of the most commonly used data sources is presented in Figure 9. On average, IUI studies used more than one method for data acquisition (M = 2.1, N = 438, SD = 1.3). Almost half of the studies (47%) performed an experiment, and almost a quarter of them (22.6%) used a questionnaire. A further forty studies (19%) presented a prototype, and 24 (11%) used an existing dataset in their research protocol.

**Figure 10:**
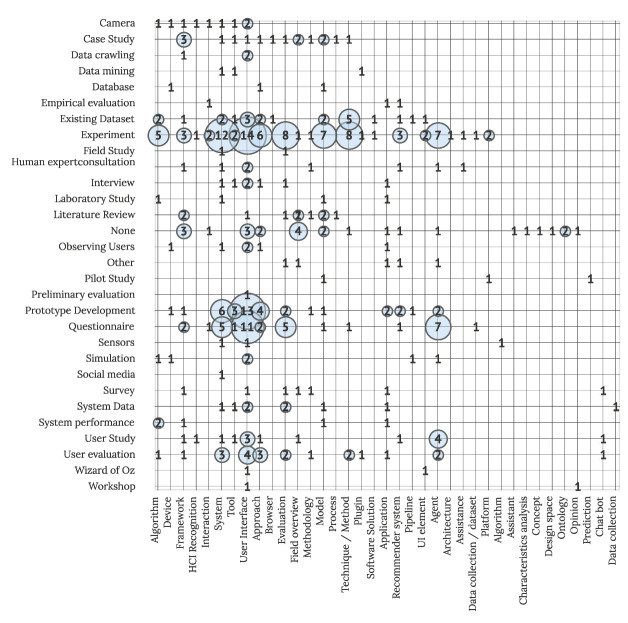
Empirical data collection methods and data sources in IUI evaluation studies (N = 211).

Figure 10 further addresses RQ2.1, visualizing empirical data collection methods and data sources in IUI evaluation studies. As observed above, an experiment is the most widely used both in proposed HCI and AI solutions in the field (e.g., studies proposing user interfaces as well as algorithms using the experiment as a method in their research protocols). Prototype development (39 studies) and questionnaire (38 studies) are widely used in studies proposing various different AI, HCI, and mixed solutions. Prototypes are mainly used in studies proposing user interfaces (13 studies), systems (6 articles), approaches (4 articles), tools (3 articles), and questionnaires are used in studies proposing user interfaces (11 studies), agents (7 studies), evaluation (5 studies), and systems (5 articles).

**Figure 11:**
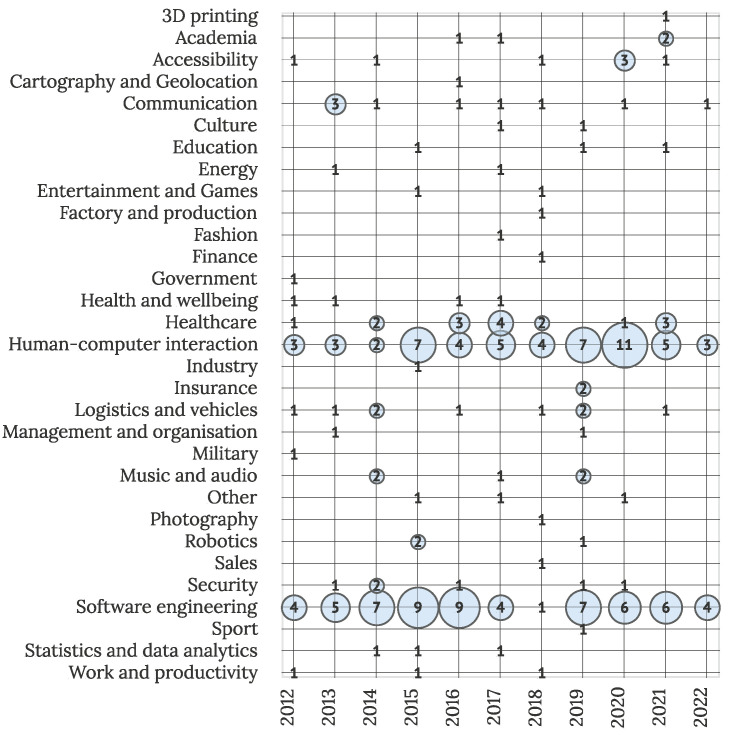
Studies by domain and year (N = 211).

The interdisciplinary field of IUI offers applications in various domains, as visible in Figure 11. Most of the observed articles are connected to SE (N = 62) and HCI (N = 54), together presenting over half (54%) of all primary studies. This suggests that the interest in the ongoing development and advancement of IUIs is the preserving theme in the field. Observing other domains, clusters of interest can be observed in healthcare (N = 16), communication (N = 9), logistics and vehicles (N = 9), and accessibility (N = 7). Although other domains were included more infrequently, their overall number suggests IUIs have various applications in different fields.

**Figure 12:**
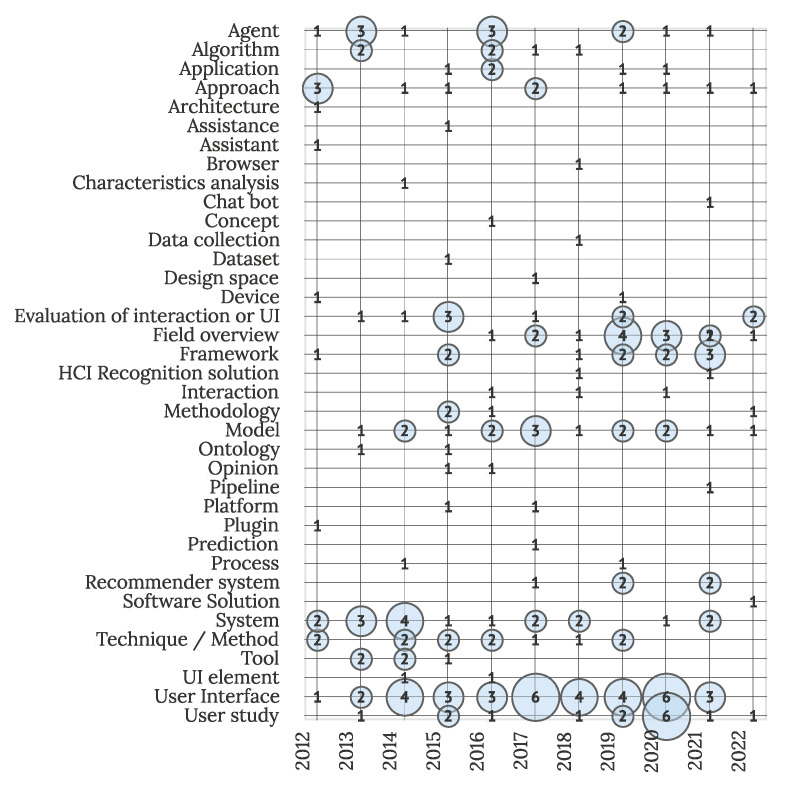
Studies by offered solution and year (N = 211).

Visualization of analyzed studies based on solutions presented to the IUI field is displayed in Figure 12. The highest focus in the field is on researching user interface–connected challenges (36 studies or 17%), with a further two focused on user interface elements. Larger clusters of studies are further focused on systems (18 studies or 9%), models (16 studies or 8%), agents (12 studies or 6%), and techniques or methods (12 studies or 6%). Dispersion of other solutions indicates a wide variety of applications where IUI is suitable.

**Figure 13:**
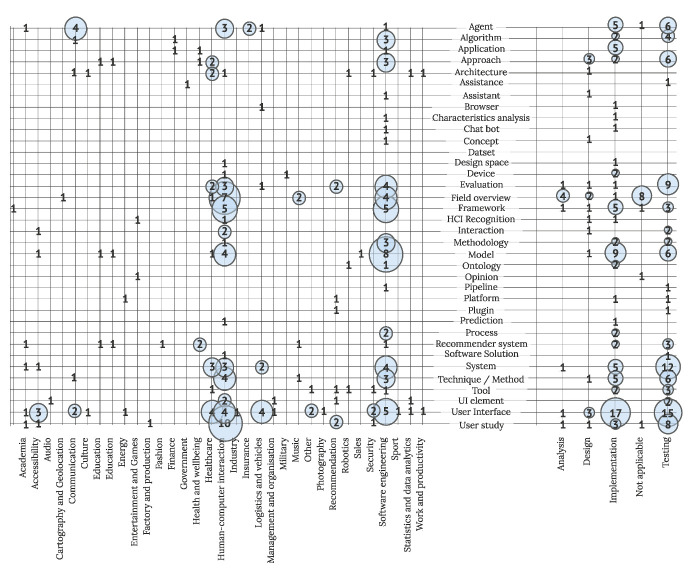
Studies by offered solution, domain, and engineering phase (N = 211).

Further insight into solutions presented to the IUI field is visualized in Figure 13, displaying overlap between proposed solutions, domains, and the engineering phase of observed solutions. Articles mainly present solutions in the testing (92 studies) and implementation (79 studies) phases of engineering. A similar ratio in favor of solutions in the implementation phase– compared to the testing phase, can be observed in user interfaces, frameworks, models, and applications. On the contrary, approaches, agents, algorithms, and systems are often presented in testing phases. Focusing on application domains, clusters of various solutions presented in the HCI and SE field can be observed, with the main proposed solutions being user interfaces, frameworks, evaluations, systems, and field overviews. User interfaces as the main proposed solutions followed by the system are the most dispersed IUI solutions applied to different application domains.

**Figure 14:**
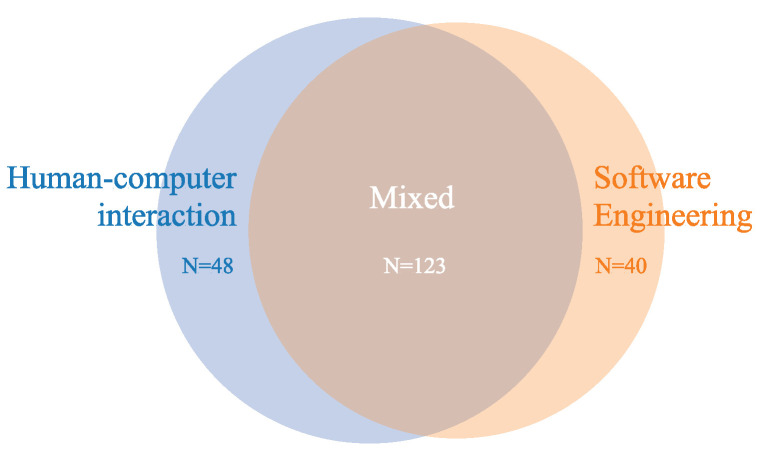
Primary studies categorized by predominant field (N = 211).

An insight into how the disciplines of HCI, AI, and SE are represented in this multidisciplinary field is provided in Figure 14. SE and AI fields were joined in one category. The majority of the studies (58% or 123 studies) included aspects of both observed disciplines, i.e., proposed an IUI solution with an accompanying algorithm along with the user interface for the proposed solution [38,39].

**Figure 15:**
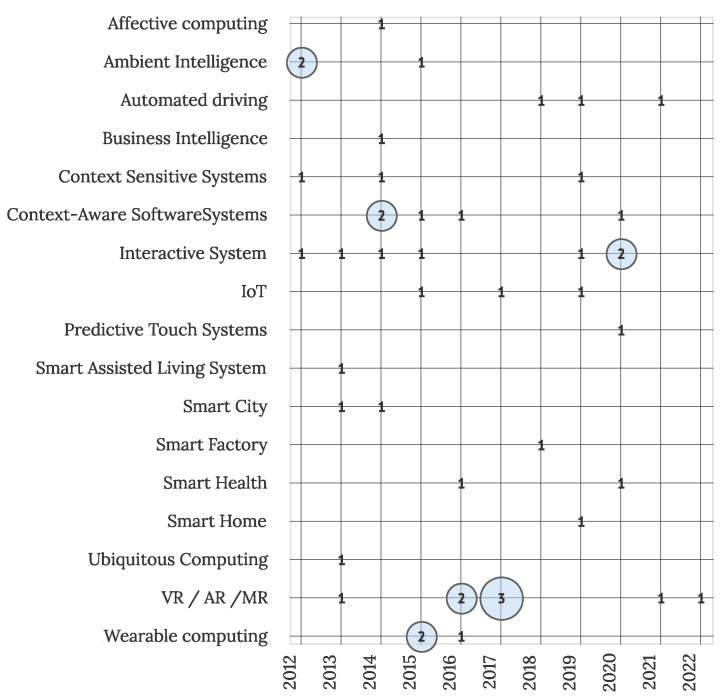
Primary studies and connection with other contemporary systems (N = 46).

Primary studies in the context of different contemporary software systems are visualized in Figure 15. Connection with other contemporary systems was observed in 46 primary studies (22% studies), with an apparent connection visible with Interactive Systems (7 studies), augmented reality (AR), virtual reality (VR) and mixed reality (MR) (8 articles), and Context-Aware Systems (5 articles). The trend of IUI application in connection with other contemporary systems was consistent in the last decade, with slight peaks visible in the last three years, displaying opportunities for connecting IUI solutions with various emerging trends.

**Figure 16:**
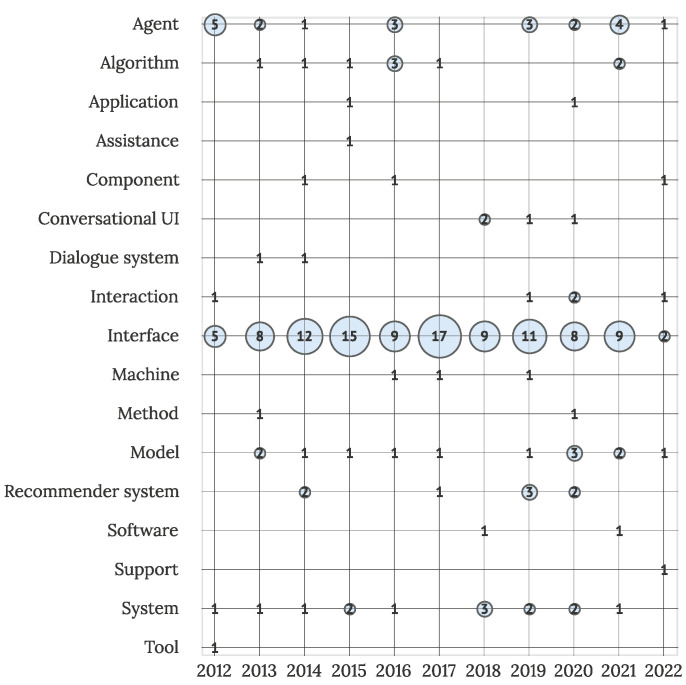
Intelligent entities in primary studies (N = 196).

To offer further insight into the debate of what is intelligent in IUIs, which was opened in [10], the primary entities considered as intelligent in each article are visualized in Figure 16. Visualization includes all the primary studies, where the determination of an intelligent entity was possible (N = 196). Half of the studies recognized interfaces as the main intelligent entities (105 studies), followed by agents (21 studies), systems as a whole (14 studies), and models (13 studies). Other entities associated with the intelligence in IUIs are component, interaction, algorithm, assistance, recommender system, conversational user interface, support, application, tool, and dialogue system.

**Figure 17:**
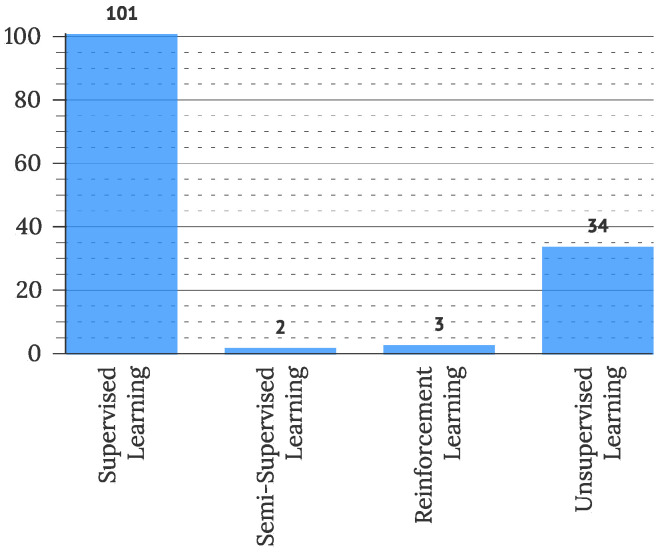
Frequency of methods used in machine learning (N = 134).

**Figure 18:**
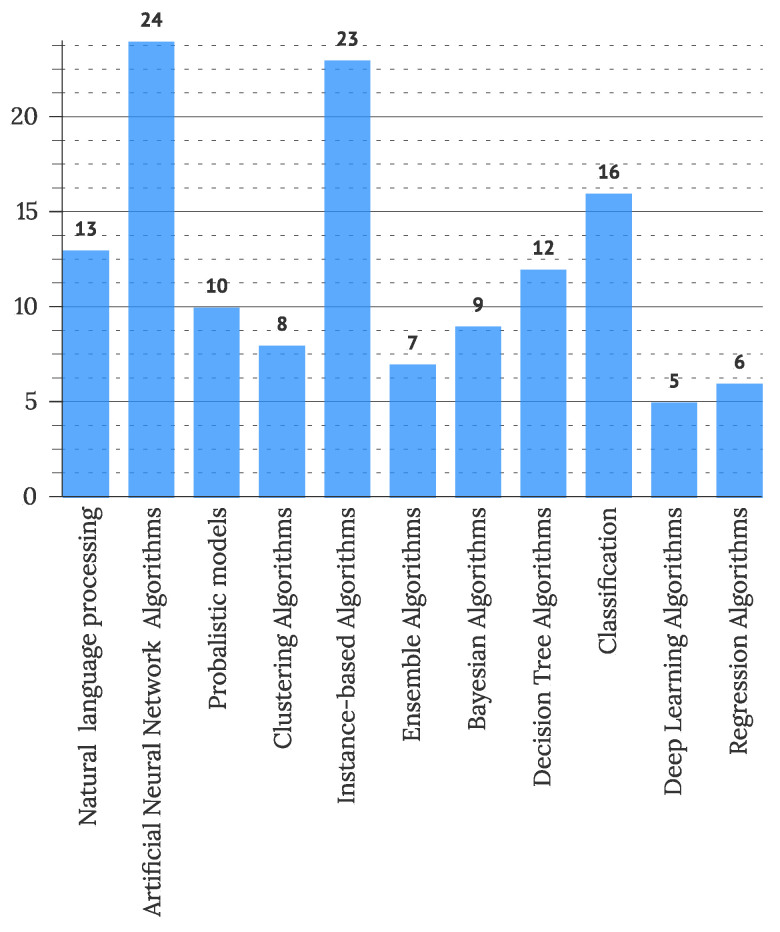
Types of used artificial intelligence algorithms (N = 117).

To address RQ2.6, used ML methods were extracted from primary studies as reported by the authors. The frequency of categorized methods is presented in Figure 17. The use of various supervised learning methods was most commonly observed (101 studies), followed by methods of unsupervised learning (34 studies). Semi-supervised (2 articles) and reinforcement learning (3 articles) were less commonly used. Further, fifty studies reported some use of AI, which could not be categorized due to the lack of information. Further categorization of used algorithms of artificial intelligence is presented in Figure 18, displaying prevalent use of artificial neural network algorithms (21% of observed studies), instance-based algorithms (20% of observed studies), and classification algorithms (14% of observed studies). Decision Tree Algorithms (10% of studies) and Bayesian Algorithms (8% of studies) were less commonly used.

### 4.3. Evaluation of IUI

**Figure 19:**
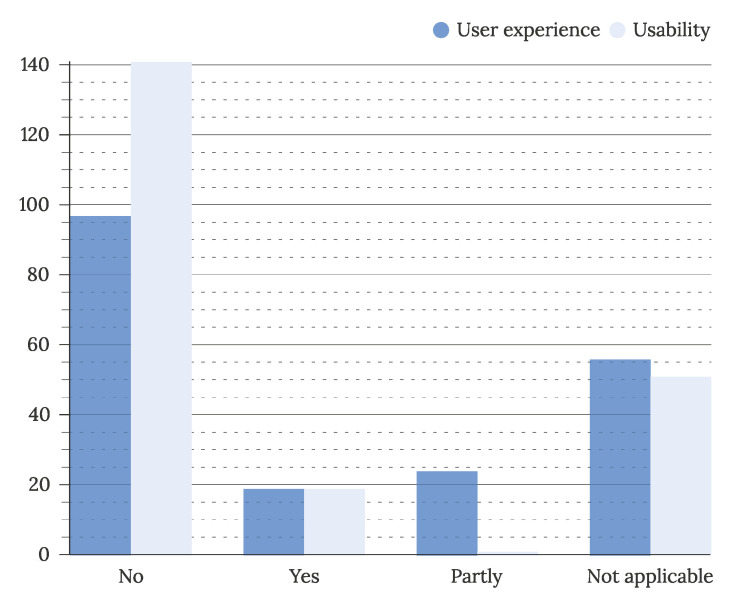
Scope of evaluation in primary studies (N = 211).

To address the evaluation of IUIs, considered in RQ3.1, the evaluation scope of primary studies is visualized in Figure 19. The majority (159 studies) of articles did not conduct usability or user experience evaluations. The evaluation was not applicable for a further 56 (27%) of studies. Of articles conducting evaluation, more (43 studies) conducted a partial (24 studies) than complete (19 articles) user experience evaluation. On the contrary, partial usability evaluation was observed in one study, whereas a further 19 studies performed a full usability evaluation. The scope of the evaluation was extracted based on terms used in the studies. Although usability is considered a part of the user experience, the terms were extracted separately.

**Figure 20:**
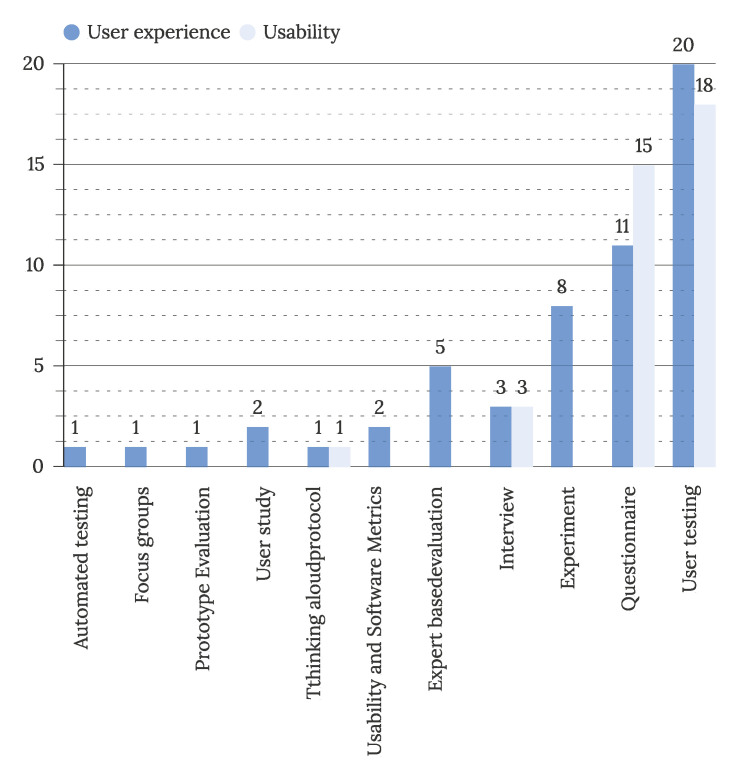
Frequency of used evaluation methods (N = 58).

Figure 20 addresses RQ3.2 with visualizing evaluation methods used for user experience and usability evaluation. The most commonly used evaluation methods for both goals are user testing (38 articles) and questionnaires (26 articles). Evaluations of IUI’s user experience also utilize experiments (8 articles) and expert-based evaluation (5 articles). Comparing automated and manual testing, the majority of evaluations are still conducted manually by experts or end-users. A combination of multiple methods is commonly used in evaluations, with an average number of 1.6 used evaluation methods (N = 32, SD = 0.7, M = 1.6) in user experience evaluations and 1.7 methods used in usability evaluations (N = 19, SD = 0.7, M = 1.7). A combination of user testing followed by a questionnaire is most often utilized (8 studies evaluating UX and ten studies evaluating usability in IUI). The number of persons included in the evaluation (users or experts) was reported in 43 studies, of which 15 evaluated the usability and another 28 evaluated user experience. The average number of users included in evaluations was higher in usability evaluations (N = 43, M = 15, SD = 50.4) compared to user experience evaluations (N = 15, M = 71.4, SD = 104.3).

**Figure 21:**
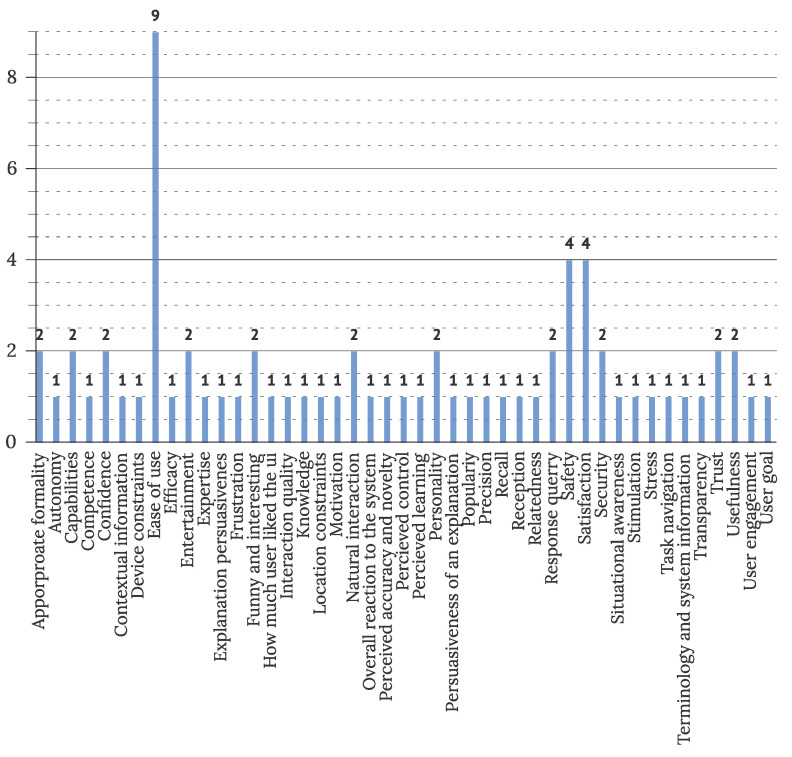
Features observed in UX evaluations of IUI (N = 20).

**Figure 22:**
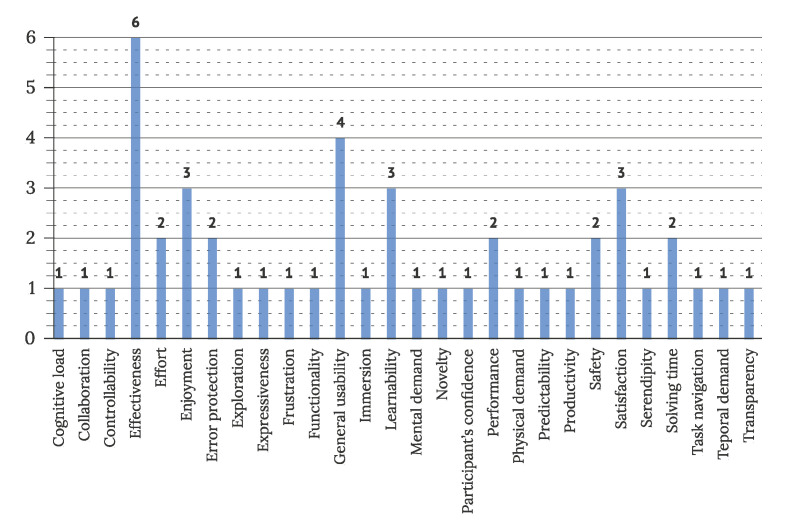
Features observed in usability evaluation of IUI (N = 18).

To address RQ3.4, characteristics and factors considered in usability and UX evaluations were extracted. Evaluated factors were reported in twenty studies observing UX and eighteen studies observing usability of IUIs. In UX evaluations, the most often observed factors were ease of use (9 articles), safety (4 articles), and satisfaction (4 articles). Figure 21 displays all noted factors and their frequency of use. In usability evaluation, effectiveness (6 articles), general usability (4 articles), enjoyment (3 articles), learnability (3 articles), and satisfaction (3 articles) were observed most commonly, as visible in Figure 22.

**Figure 23:**
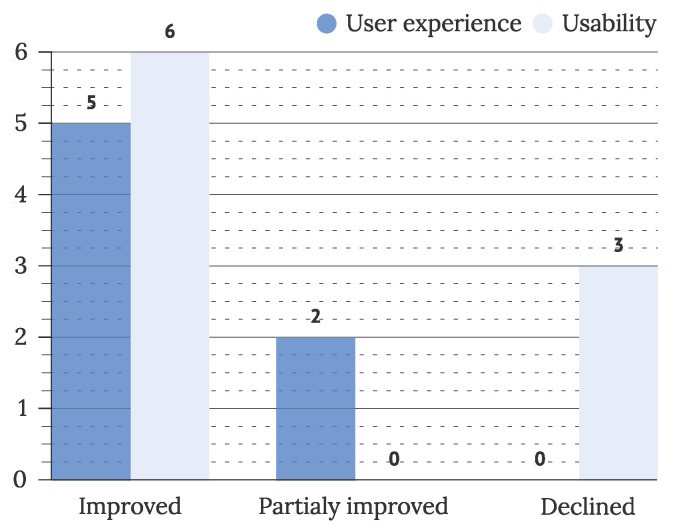
Improvement of usability and UX (N = 16).

The overall results of IUI evaluations concerning improvements of observed IUIs are presented in Figure 23. Improvement was analyzed and reported in a few studies (N = 16), with most studies reporting improvement (5 studies) or partial improvement (2 studies) in measured user experience and improvement in measured usability (6 studies). Three studies (S60, S165, and S207) reported worse measurements of observed usability factors after their interventions.

## 5. Discussion

### 5.1. Evaluation Insight and Challenges

The results of this study indicate higher interest in presenting newly developed ideas in the field of IUI with a limited evaluation of IUI conducted. Non-uniform use of usability and UX factors was observed, as well as issues reported in related fields [30]; usability and UX are not considered together but separate, with the evaluation of one seemingly excluding the other in the published studies. Evaluated factors are not clearly categorized as usability and UX in the studies, with safety, satisfaction, and transparency being sometimes considered as UX and sometimes as usability factors.

The need for standardized testing tools and evaluation protocols, identified in [40], still presents a research opportunity that will need further addressing as IUIs become widely adopted in everyday life. The issues with the shortage of meaningful standardized measures are still present, as the extraction criteria, based on evaluation goals, measures, and baseline, expected, and observed values were dropped due to the shortage of information in most of the published studies. We note that the main usability factors, important in IUI adaptation, presented by [41] (spatial stability, locality, accuracy, predictability, interaction frequency, task complexity, and average interaction costs) still have no universal evaluation methods and metrics, or they are not being reported in the studies.

The challenge of proving the benefits IUIs introduce to the interaction with the user, which was presented in [42], was also addressed in our work. We confirmed that improvement of HCI interaction after presenting IUIs was a trend observed in the last decade. Most evaluations of usability and UX (81% of studies) presented partial or recognizable improvement in observed metrics. However, the number of evaluations conducted on presented IUI studies is yet to rise. Triangulation of observed evaluation methods is not yet used as the evaluation norm, though most of the observed studies use up to two methods in their evaluation, mainly user testing and questionnaires. The lack of cost-effective IUI evaluation guidelines, pointed out by [40], is one of the possible reasons. Observed evaluation tools, such as SUS, QUIS, NASA Task-Load index, and situation awareness rating technique, could offer some resolution to this issue.

We further identified a research gap in the evaluation of proposed IUI solutions in terms of accessibility. Seven observed studies were published in the accessibility domain, whereas only three evaluated usability (S21, S35, and S209) and one evaluated UX (S38).

### 5.2. Implication to the Debate of Intelligence in IUI

This study also offers some contributions to the debate on what is intelligent in user interfaces, which was disclosed by [10], who presented that mainly, artificial intelligence is included in IUIs in the form of intelligent agents, algorithms, tools, and assistants. We further show that IUIs mostly utilize artificial neural networks, instance-based algorithms, classification, natural language processing, and probabilistic algorithms to introduce artificial intelligence in the IUI solutions. Furthermore, a strong trend of using machine learning methods is observed, with more than half of the studies presenting some form of machine learning in their solutions, with the prevalent use of supervised learning (101 articles). We confirm the observed diversification of intelligent entities (Figure 16) presented in IUI articles, which was earlier recognized by [10]. However, the central intelligent entity, recognized in IUI studies, remained the user interface in all observed years. Performed definition analysis confirmed the correlation between adapt* terms and IUIs, Volkel [10] presented. Two definitions connected IUI directly with the natural language processing and less with the general AI area. An observed narrower definition could result from an ad-hoc understanding of IUIs, as half of the observed primary studies (N = 134) included ML methods, with thirteen studies using the natural language processing methods.

### 5.3. Limitations

As expected, this work is bound by limitations due to a few months long timespan of reviewing the studies and conducting the classification. A large number of identified prospective publications were manually assessed as suitable or unsuitable for usage in the future. As articles were manually screened in the span of a few months, a possibility of accidental, wrongful rejection of an important article exists. This study is focused on intelligent user interfaces, and its finding cannot be generalized to the HCI domain and are not directly transferable to similar fields (e.g., adaptive user interfaces). A possibility of human error should also be considered. The limitation of wrongful classification was mitigated with the consultation of all involved researchers for marginal cases. Limitations regarding the analysis of the definitions are threefold; (i) *time*—only the definitions cited in the selected articles from the last decade were included in our sample, effectively excluding previous definitions that might have been used in earlier studies, published in the area; (ii) *quality*—the quality of used definitions varied from the dictionary and ad-hoc definitions used to place the IUI in the context of short conference articles to analytical definitions, presented in other, more complex studies; (iii) *scope*—performed analysis was conducted only on the selected sample of academic articles, and although some studies cited dictionary definitions, other potential primary sources were not included in this study.

### 5.4. Threats to Validity

The major threats to our study’s validity are identifying relevant primary research, data extraction, and classification. First, the chosen keyword and query may have excluded several studies (e.g., studies primarily using the term adaptive interfaces). However, in order to include as many relevant, recent studies as possible, our search included six digital libraries. Secondly, as the observed research field is decades old and the interest of this study was in the most recent trends, we removed a significant number of articles based on the year of publication. Consequently, some of the fundamental and groundbreaking research was excluded. Due to the vast range of fields, intercepting in the IUI field, and the diverse quality and complexity of the observed studies, the data extraction and classification process was challenging. To improve the validity of the initially proposed classification scheme, we conducted a pilot study and improved the scheme over various iterations. For easier comparison of findings with other studies, schemes used in related work were also considered while conducting the pilot study.

## 6. Conclusions

This work adds to the body of knowledge in the multidisciplinary field of intelligent user interfaces, mainly connecting HCI and AI. An unbiased, objective, and systematic overview of the trends in the IUI field in the last decade is presented. From a total of 9849 studies, after introducing narrowing filters, we selected 211 articles and extracted their characteristics in our mapping.

The following findings were obtained by our analysis. The scientific interest in IUI solutions is active and followed by a large body of research, which reached a recent peak in 2019. Studies mainly include mixed aspects of the HCI and AI areas. The research is primarily quantitative, with the experiment being the main research type. Similarly, experiments, prototype development, and questionnaire are the main data acquisition methods observed in the primary studies. The main contributions in published studies are, in a larger part, user interfaces and systems. Solutions most often belong to HCI and SE domains, later followed by healthcare. A wide number of active domains in which IUIs are applied is observed. More than half of the solutions used ML techniques and algorithms; most studies observed the utilization of supervised learning. The analysis of AI algorithms, artificial neural network algorithms, and instance-based algorithms, classification, and natural language processing were the most prevalent methods.

The following opportunities were recognized for future research: evaluation of IUI lacks guidelines for testing IUI-specific characteristics along with cost-effective, standardized methods that would offer fast and widely applicable evaluation of proposed solutions. Similarly, the analysis of metrics best applicable for IUI evaluation presents an opportunity for future research. Publications of lessons learned in the form of experience articles with this challenge. Opportunities for joint evaluation of UX and usability are observed, as they could offer some wider insight into the human–computer interaction with IUIs. Applications of IUIs in the accessibility domain are still sparse, although they have the opportunity to improve human–computer interaction for persons with disabilities. The variation of domains where IUIs have been suggested in recent years suggests the possibilities of their diverse application in other fields and everyday situations.

## Figures and Tables

**Table 1:** Overview of related studies.

Study	Terms Used	No. of Studies	Method	Search Query	Databases
[6]	AUI, IUI	43	SMS	(Intelligent User Interface OR IUIs OR Adaptive User Interface) AND (Tech* OR Desig* OR Meth*)	ACM Digital Library, IEEEXplore, Springer Link
[10]	IUI	1111	Meta-Analysis	“intelligent”	ACM IUI conference
[7]	AUI, IUI	165	SLR	“(((((((((((Dynamic UI Design) OR Plasticity) OR Adaptive) OR Adaptation) OR Adaptability) OR Adaptivity) OR Universal Usability) OR ubiquitous) OR Inclusive Design) OR pervasive) AND User Interface Design)”	ACM Digital Library, Cambridge Journals, EBSCO Host, IEEEXplore, Oxford Journals, Sage Journals, Saudi Digital Library, ScienceDirect, SciSearch, Scopus, Springer Link, Web of Knowledge, Wiley
[29]	Explainable AI	12,701	Literature analysis	Manual selection expanded with keyword search for “intelligible”, “interpretable”, “transparency”, “glass box”, “black box”, “scrutable”, “counterfactuals” and “explainable”	Google Scholar, Scopus
[9]	HCII, IUI	454	SMS	“intelligent interaction” OR “intelligent user interface”	ACM Digital Library, IEEEXplore, ScienceDirect, Scopus, Web of Science
[8]	AUI and adaptable systems	63	Systematic and empirical literature review	User-centered evaluations of personalized systems	ACM Digital Library, ERIC, Easy-D, IEEEXplore, INSPEC, PsycInfo, ScienceDirect, Scopus, Web of Science
[30]	Natural UI	56	SMS	(“natural user interface*” OR “natural interface*” OR“natural user interaction*” OR “natural user communication*” OR “natural communication”) AND intervention (“tool” OR “framework” OR “technique” OR “method”OR “model” OR “process” OR “guideline” OR“pattern” OR “metric” OR “approach” OR “inspection”OR “principle” OR “aspect” OR “requirement” OR“heuristic” OR “methodology” OR “mechanism”) AND outcome (“Usability evaluation” OR “Usability assessment”OR “Usability improvement” OR “ux evaluation” OR “ux assessment” OR “ux improvement” OR “userexperience evaluation” OR “user experience assessment” OR “user experience improvement”)	ACM Digital Library, Engineering Village, IEEEXplore, Scopus, ScienceDirect
[5]	AUI, IUI, Multi-modal UI, Smart UI	151	SLR	“smart user interface” OR “Intelligent user interface” OR “adaptive user interface” OR “context-sensitive user interface” OR “multimodal user interface” OR “smart human computer interface” OR “Intelligent human computer interface” OR “adaptive human computer interface” OR “context-sensitive human computer interface” OR “multimodal human computer interface” OR “smart human machine interface” OR “intelligent human machine interface” OR “adaptive human machine interface” OR “contextsensitive human machine interface” OR “multimodal human machine interface” OR “smart graphical user interface” OR “Intelligent graphical user interface” OR “adaptive graphical user interface” OR “context-sensitive graphical user interface” OR “multimodal graphical user interface” OR “IUI”	Scopus, Web of Science

SMS: Systematic mapping study, SLR: Systematic literature review.

**Table 2:** Research questions.

	Research Question
RQ1	What have been the trends and demographics of the literature within the field of IUI? The following subquestions were formulated:
RQ1.1	What is the annual number of publications in the IUI field?
RQ1.2	What demographic and literature trends can we observe in the last ten years in the IUI field?
RQ1.3	Which venues are the main targets of the IUI research?
RQ1.4	Which are the top-cited studies in the last decade in the IUI field?
RQ1.5	Contributors from which countries are the most active in the IUI field (based on the affiliated institutions)?
RQ1.6	How have IUI’s been defined in the last decade?
RQ2	What has been the research space of the literature within the IUI field in the last decade? The following subquestions were formulated:
RQ2.1	What methods are used to conduct research in the IUI field?
RQ2.2	Which domains are connected to IUI publications?
RQ2.3	What kind of solutions are offered in the contributions to the IUI field?
RQ2.4	Are proposed solutions used in other contemporary systems?
RQ2.5	Which intelligent entities are subjects of the IUI research?
RQ2.6	How is artificial intelligence included in the IUI field?
RQ3	How is the evaluation of intelligent user interfaces conducted? The following subquestions were formulated:
RQ3.1	How do researchers evaluate IUI (UX, usability)?
RQ3.2	Which evaluation methods are used?
RQ3.3	How many users or experts are included in evaluations?
RQ3.4	What factors are considered in IUI evaluations?
RQ3.5	Do proposed IUI solutions improve usability and user experience?

**Table 3:** Articles retrieved from selected digital libraries using the presented query.

Library	No. of Papers
ACM Digital Library	256
IEEEXplore	1852
ScienceDirect	546
Scopus	4205
Web of Science	2989
Together	9849

**Table 4:** Inclusion and exclusion criteria.

	Criteria	Description
I1	Field	Include studies addressing intelligent interaction or intelligent user interfaces.
I2	Language	Include studies written in English.
I3	Literature type	Include studies published in peer-reviewed journals, conference proceedings, or magazines.
I4	Year	Include literature published in 2012 and later.
E1	Research area	Exclude non-computer science or non-human–computer interaction literature.
E2	Duplicates	Exclude any duplicated studies found in multiple databases.
E3	Field	Exclude studies outside of the scope of IUI.
E4	Comprehensiveness	Exclude papers less than two pages long that do not provide enough information about the study conducted.
E5	Availability	Exclude papers not accessible electronically.

**Table 5:** Steps in screening and selection of the relevant literature.

Step	Activity	No. of Papers
I	Query execution in digital libraries (I1–I4 applied)	1036
II	Removing duplicates (E2 applied)	607
III	Screening by article and abstract (applied E1, E3)	376
IV	Screening with quickly reading the manuscripts (applying E4, E5)	211

**Table 6:** Final classification scheme.

	Variable	Possible Answers
EC1	Research type	Evaluation research, Experience paper, Literature review, Longitudinal study, Opinion paper, Philosophical Papers, Solution proposal, Validation research
EC2	Publication type	Conference paper, Journal article
EC3	Standpoint	Human–computer interaction, Artificial intelligence and Software Engineering
EC4	Methodology	Quantitative, Qualitative, Mixed, Non-empirical, Not applicable
EC5	Primary research strategy	Case Study, Experiment, Exploratory study, Feasibility study, Field Study, Grounded Theory, Literature review, Other, Prototype, Survey, User Study
EC6	Data acquisition methods	Interview, Observing users, Prototype development, Questionnaire, Systematic literature review, Wizard of Oz, Human expert consultation, Case study, Simulation, User evaluation, Laboratory study, Literature review, Sensors, Empirical evaluation, Camera, Database, Data crawling, Mobile phone sensors, Pilot study, Experiment, System data (user behavior), System performance metrics, Survey, Workshop, Existing dataset, Within subjects study. User study, Linguistic analysis, Field study, Data mining, Manual measurements, Field test, Social media, Other
EC7	Sofware type	Camera, Case study, Data crawling, Data mining, Database, Empirical evaluation, Dataset, Experiment, Field study, Human expert consultation, Interview, Laboratory study, Literature review, Method, None, Observing users, Other, Pilot study, Preliminary evaluation, Prototype development, Questionnaire, Sensors, Simulation, Social media, Survey, System data, System performance, User evaluation, User study, Wizard of oz, Workshop
EC8	Engineering phase	Analysis, Design, Implementation, Testing, Not applicable
EC9	Domain	3D printing, Academia, Accessibility, Cartography and geolocation, Communication, Culture, Education, Energy, Energy, Entertainment and games, Factory and production, Fashion, Finance, Government, Health and wellbeing, Healthcare, Human-computer interaction, Industry, Insurance, Logistics and vehicles, Management and organization, Military, Music and audio, Not applicable, Other, Photography, Recommendation, Robotics, Sales, Security, Software engineering, Sport, Statistics and data analytics, Work and productivity
EC10	Intelligent entity	Interface, Machine, Support, Recommender system, System, Interaction, Agent, Tool, Application, Environment, behavior, Interface agent, Assistance, Software, Tutoring system, Algorithm, Method, Control, Robot, Multimedia interface, Information system, Component, Selection, Technique, Intelligent assistant, Not applicable, User model, Conversational UI, Other model, Dialogue system
EC11	Solution	Agent, Algorithm, Application, Approach, Architecture, Assistance, Browser, Dataset, Device, Evaluation, Field overview, Framework, HCI Recognition, Interaction, Methodology, Model, Pipeline, Platform, Plugin, Process, Recommender system, Software Solution, System, Technique/Method, Tool, UI element, User Interface
EC12	IUI definition	Definition of IUI and the source used (if available)
EC13	Part of contemporary system	Affective computing, Ambient Intelligence, Automated driving, Business Intelligence, Context Sensitive Systems, Context-Aware Software Systems, Interactive System, Internet of Things, Predictive Touch Systems, Smart Assisted Living System, Smart City, Smart Factory, Smart Health, Smart Home, Ubiquitous Computing, VR/AR/MR, Wearable computing
EC14	Artificial intelligence methods and algorithms	Open description
EC15	UX evaluation	Yes, No, Partly, Not applicable
EC16	Evaluated factors of UX	Appearance, Perceptions, Performance, Availability, Overall satisfaction, Time, Efficience, Effectivenes, Productivity, Error safety, Accuracy, Costs, Ease of use, Other
EC17	UX Evaluation method	A-B testing, Automated testing, Experiment, Expert–based evaluation, Focus groups, Interview, None, Not applicable, Questionnaire, Thinking aloud protocol, User study, User testing
EC18	UX improved	Yes, Yes – partly, No, Not applicable
EC19	Usability evaluation	Yes, No, Partly, Not applicable
EC20	Usability evaluation method	Survey, User Testing, Heuristic Evaluation, Interview, “thinking aloud protocol”, Usability Metrics/Software Metrics, Automated Evaluation via Software Tool/Software, Cognitive Walkthrough, Prototype Evaluation, “Other, None, Model–based evaluation, Review based evaluation, Feature inspection, Pluralistic Walkthrough, Formal Usability Inspection, Questionnaire
EC21	Evaluated factors of usability	Learnability, Appropriateness recognizability, Operability, User error protection, User interface aesthetics, Accessibility, Other (open description)
EC22	Usability improved	Yes, Yes—partly, No, Not applicable
EC23	Evaluation tools	System usability scale (SUS), NASA Task-Load index, Questionnaire for User Interaction Satisfaction (QUIS), Other (open description)
EC24	Number of test users	Open input
EC25	Type of testing	Manual, Automatic

**Table 7:** The classification scheme for IUI definition analysis.

	Variable
EC1	Reference
EC2	Scope
EC3	Distinctive characteristics
EC3	Performed actions
EC4	Mentioned technologies
EC5	Adaptation to
EC7	What is adapted

**Table 8:** Most represented journals and conferences (number of included articles > 1).

Journal	No. of Papers
ACM Transactions on Interactive Intelligent Systems 2020	9
Interacting with Computers	3
IEEE Access 2022	2
**Conference**	**No. of Papers**
ACM International Conference on Intelligent User Interfaces (and companion)	44
Lecture Notes in Computer Science (including subseries)	19
CEUR Workshop Proceedings	10
Conference on Human Factors in Computing Systems	6
ACM International Conference Proceeding Series	5
Communications in Computer and Information Science	3
ICAART—11th International Conference on Agents and Artificial Intelligence	3
ICMI—International Conference on Multimodal Interaction	3
Advances in Intelligent Systems and Computing	2
ICEIS—International Conference on Enterprise Information Systems	2

**Table 9:** Top ten most cited articles.

Title	Journal/Conference	Year	No. of Citations	Average Yearly Citations
Hessian Regularized Support Vector Machines for Mobile Image Annotation on the Cloud	IEEE Transactions on Multimedia	2013	98	10.9
Frequence: Interactive Mining and Visualization of Temporal Frequent Event Sequences	ACM International Conference on Intelligent User Interfaces	2014	82	10.3
Measurable Decision Making with GSR and Pupillary Analysis for Intelligent User Interface	ACM Transactions on Computer Human Interaction	2015	62	8.9
Rhema: A Real-Time In-Situ Intelligent Interface to Help People with Public Speaking	ACM International Conference on Intelligent User Interfaces	2015	61	8.7
Exploring vibrotactile feedback on the body and foot for the purpose of pedestrian navigation	ACM International Conference Proceeding Series	2015	55	7.9
Personalized explanations for hybrid recommender systems	ACM International Conference on Intelligent User Interfaces	2019	52	17.3
Westland row why so slow? Fusing social media and linked data sources for understanding real-time traffic conditions	ACM International Conference on Intelligent User Interfaces	2013	48	5.3
Cohort Comparison of Event Sequences with Balanced Integration of Visual Analytics and Statistics	ACM International Conference on Intelligent User Interfaces	2015	45	6.4
Both complete and correct? Multi-objective optimization of touchscreen keyboard	Conference on Human Factors in Computing Systems	2014	37	6.6
User-centered visual analysis using a hybrid reasoning architecture for intensive care units	Decision Support Systems	2012	34	3.4

**Table 10:** Top countries regarding the contribution to the literature.

Country	No. of Papers	%
USA	56	27%
Germany	25	12%
United Kingdom	13	6%
China	9	4%
Japan	8	4%
France	7	3%
Ireland	6	3%
Belgium	5	2%
Brazil	5	2%
Israel	5	2%
Italy	5	2%
Australia	5	2%
Austria	5	2%
Russia	5	2%
Spain	5	2%

## Data Availability

Not applicable.

## Supplementary Materials

The following are available at: https://www.mdpi.com/article/10.3390/s22155830/s1, Studies included in the systematic mapping study.

## Abbreviations

The following abbreviations are used in this manuscript:

AI	Artificial intelligence
AUI	Adaptive user interface
HCI	Human–computer interaction
IUI	Intelligent user interface
ML	Machine learning
SE	Software engineering
SLR	Systematic literature review
SMS	Systematic mapping study
UI	User interface
UX	User experience

## Appendix A. Extracted Definitions of IUI

**Table A1:** Definitions analysis: extracted definitions, scope, and technology described.

	Study	Extracted Definition	Scope		Technology				
			**Field, Domain**	**User Interface**	**Ad-Hoc**	**Technology-Focused**	**Includes ML**	**Includes AI**	**Intelligent**
D1	S91	“The term intelligent hereby describes the ability of a user interface to extensively adapt to a usage context, i.e., to a specific user, task and available tools.”							
D2	S92	“Intelligent UI adaptation allows the application to act more like a human and consequently, more intelligently.”		x					
D3	S199	“… and intelligent and adaptive means to implement the proper response techniques. Furthermore, the intelligent interface can learn about the subject’s lifestyle and adapt the interaction style/mode accordingly.”		x					
D4	S138	“Intelligent User Interfaces(IUI) are the human-machine interfaces that has the objective of improving the effectiveness, naturalness and the efficiency of interaction (human machine interactions)by reasoning(involving (AI)artificial intelligence methods), representing and modeling the users, tasks, device and context.”		x					
D5	S189	“Adapting to human behavior and the underlying intentions is increasingly important in developing intelligent user interfaces in different fields of application”		x					
D6	S56	“An intelligent user interface (IUI) should be able to adapt its behavior to different users, devices and situations.”		x					
D7	S6	“An intelligent user interface is a UI that contains some perspective of artificial intelligence in computing. This makes the interface more comprehensive, customizes and guides the interaction.”		x					
D8	S118	“An intelligent user interface, in general, focuses on the interaction between machine intelligence and human intelligence.”		x					
D9	S29	“…an IUI is typically based on the computer-aided knowledge of a specific subject area and/or the user model. Due to that the interface can better understand the user’s needs and personalize or support the interaction. In other words, the IUI may be interpreted as follows: it is a software tool that has an intelligent interface and uses intelligent techniques when interacting with the user. The IUI might be based on some user models, or on knowledge of the system functionality, or on procedures helping the user.”…“The generation of interfaces called “intelligent” is designed to provide the user with additional capabilities, including adaptability, customization and assistance in solving problems. The implementation of the intelligent user interface should be an intelligent agent or intermediary between the user and a particular computer application that implements approaches and methods to support communication with the user.”		x					
D10	S52	“Based on published researches, one can list the functions thatare closely related to the notion of the intelligent interface [35,43]: 1. analysis of the user’s actions; 2. a (speech-based) dialogue system; 3. adaptability, i.e., the system can change the pattern of its behavior, according to the situation.”		x					
D11	S56	“Besides, these interfaces belong to software systems that are capable of adapting themselves to their users.”				x			
D12	S34	“…by their capability to adapt at run-time and make several communication decisions concerning ’what’, ’when’, ’why’ and ’how’ to communicate.” Therein, they relate to an earlier view of a UI communicating concepts to the user.”		x					
D13	S35	“…focusing on interfaces that require intelligent technologies to bring them to fruition [44]. These include natural language techniques in interfaces, interfaces for intelligent learning systems, new methods for recognizing gestures and attention, among others.”				x			
D14	S20	“Generally, an intelligent user interface means that the computer side has advanced understanding of the environment, which enables the interface to better understand the user’s needs and to personalize or lead the interaction.”				x			
D15	S57	“…human-machine interfaces that aim to improve the efficiency, effectiveness, and naturalness of human-machine interaction by representing, reasoning, and acting on models of the user, domain, task, discourse, and media—intelligent user interfaces (IUIs), use artificial intelligence (AI), human-computer interaction (HCI), software engineering (SE) and other techniques to promote more natural and usable human-machine interaction.”		x					
D16	S29	“In addition, however, intelligent interfaces promise to provide additional features, such as [45]: • recognition of inaccurate, ambiguous and/or partial multimodal input of information (acquiring and processing information in the system using various devices such as mouse, keyboard, microphone); • creating a coherent, unified and understandable multimodal representation; • fully or partially automatic problem solving; • interaction management (problem-solving, customization, interface adaptation) through representation, inference, and use of user model, domain model, task model, and context model. In other words, “intelligence” of the user interface can be interpreted as some subroutine-agent”		x					
D17	S172	“In this paper we discuss the key characteristics of Intelligent User Interface (IUI) for cloud manufacturing, i.e., naturality, smart mobility, self-configuration, and flexible customization.”		x					
D18	S84	“In this paper, we use the term IUIs to refer to interfaces in which the system tries to interpret the user’s intent.”			x				
D19	S34	“…intelligent user interfaces “are human-machine interfaces that aim to improve the efficiency, effectiveness, and naturalness of human-machine interaction by representing, reasoning, and acting on models of the user, domain, task, discourse, and media (e.g. graphics, natural language, gesture).” Their definition entails the ability to think and to understand language and gestures (linguistic and physicalkinaesthetic intelligence). Also, it defines the goal or purpose of technological intelligence– efficiency, effectiveness, and naturalness of interaction.”		x					
D20	S171	“Intelligent User Interfaces (IUI) are technologies intended to mediate between human users and machine reasoners (e.g., [46,47]). Like cognitive architectures, they are inspired by insights from experimental psychology, but in this case the focus is on insights into the functioning of the human perceptual system rather than internal reasoning mechanisms”				x			
D21	S139	“Intelligent user interfaces are being proposed as a means to make systems individualized or personalized, thereby increasing the system’s flexibility and appeal…”			x				
D22	S151	“Intelligent user interfaces have the goal of dynamically adapting to the needs of a user as they interact with an information system.”			x				
D23	S115	“Intelligent User Interfaces refers to the study and use of the two major fields of the Computer Science that are Human Computer Interaction (HCI) and Artificial Intelligence (AI). HCI provides efficient user interfaces designing techniques and AI is used to automate or to build intelligence in those interfaces. Basically the term IIUIs suggests that an interface or interface like system, which is interacting with the user, must generate some output that the user considers it an intelligence, e.g., if the user don’t know how to copy files in the windows operating system then in the windows help there must be assistance available for this when user searches for this, if user clicks the wrong button then an automatic message must be appeared for the help of the user, if the user has previously selected some options on the interface based on those options the system must understand the interests of the user and generates output according to it.”	x						
D24	S148	“Intelligent user interfaces were proposed as a means to make systems more adaptive to people, because end-users often have to tackle information overflow or cognition overload problems when facing complex systems.”			x				
D25	S87	“IUIs are interfaces with adaptive, communication and problem-solving capabilities to help the user in an intelligent manner.”		x					
D26	S57	“IUIs use adaptation techniques to be “intelligent/ adaptive”, i.e., the adaptivity is built into the system. Thus the system with an IUI automatically adapts some aspects considering the current context of use.”			x				
D27	S153	“Many also are the reasons why the computer application interfaces can be called intelligent: interfaces that have knowledge about the functionality of the application, interfaces that have knowledge of user preferences, self-adaptive interfaces, i.e., the interfaces that on the basis of interaction with the user can adapt themselves to his needs, interfaces processing natural language and using semantic analysis.”		x					
D28	S35	“Research in Intelligent User Interfaces describes a broad class of system types that apply Artificial Intelligence techniques to different aspects of Human-Computer Interaction.”			x				
D29	S28	“The foundations of adaptive or intelligent user interfaces (AUI or IUI) are presented, three core domains being focused upon: Artificial Intelligence (AI), User Modelling (UM) and Human–Computer Interaction (HCI).”			x				
D30	S52	“The intelligent user interface allows to increase the controlled object autonomy level, as well as contributing to naturalness and ergonomics of human-machine interaction, allowing the user to take advantage of convenient and/or natural ways of interaction in accordance with his needs. The former aspect, namely, increasing the level of autonomy of the system, is important for complex robotic systems that function in information uncertainty conditions, the latter one (improving ergonomics and naturalness of the interface)—is important when the robotic system interacts with humans (social robotics, assistive robotics, exoskeletons, etc.).”		x					
D31	S8	“The need for intelligence in UIs has also driven the research and development of new AI methods and algorithms, enabling the development of human–computer intelligent interactions (HCII) and IUIs.”	x						
D32	S138	“… the systems which can mimic the human dialogue, and possibly the adaptive interfaces.”			x				
D33	S49	“The term “intelligent human-machine interface” is understood as interface that uses artificial intelligence technologies [43] Intelligent user interfaces allow increasing the autonomy of the device and contribute to the naturalness and ergonomics of human–machine interaction; thus, the user is free to use the ways of interaction that are convenient, or natural for certain situations.”		x					
D34	S87	“These interfaces belong to a type of intelligent systems that are capable of self-adapt to users with different health problems, this is possible through the determination of behavior characteristics that distinguishes each user from another.”			x				
D35	S115	“These interfaces change their behavior according the real time scenario on which it is implemented.”			x				
D36	S66	“This component adapts and renders elements of an interface through the use of ML algorithms by understanding the user’s goal.”			x				
D37	S101	“To coordinate the different professionals, trusted sources and systems that are dynamic and able to customize responses rapidly are needed to provide real-time information New intelligent user interfaces promise to increase the coordination quality and reduce the time frame to do this.”			x				
D38	S204	“To summarize, instead of the user adapting to an interface, an IUI can adapt to the user and its environment. The IUI tries to determine the needs of an individual user and attempts to maximize the efficiency of the communication. This approach is similar to an agent development toolkit according to specifications for interoperable agent-based systems.”		x					

Mark “x” is used to indicate characteristics extracted from definitions.

**Table A2:** Definitions analysis: Descriptive characteristics of IUI.

	Adaptation/Adaptabilty	Personalization	Ability to Reason	Runs at Runtime	Ability to Mimick	Ablity to Understand	Ability to Decide	Ergonomic	Natural	Customizable	Ability to Represent	Assistance	Ability to Reason	Ability to Communicate	Comprehensive	Autonomous	Ability to Analyze User’S Actions	Ability to Learn	Ability to Recognize	Ability to Model	Intelligence	Ability to Problem Solve
D1	x																					
D2	x																				x	
D3	x																	x			x	
D4			x								x									x		
D5	x																					
D6	x																					
D7															x							
D8																						
D9	x									x		x										
D10	x																x					
D11	x																					
D12				x			x							x								
D13																			x			
D14		x				x																
D15											x		x									
D16	x									x	x								x			x
D17										x												
D18																						
D19											x		x									
D20			x																			
D21		x																				
D22	x																					
D23																					x	
D24	x																					
D25	x											x										x
D26	x																					
D27	x																					
D28																					x	
D29	x																			x		
D30								x								x						
D31																						
D32	x				x																	
D33								x	x							x						
D34	x																					
D35	x																					
D36	x																					
D37												x										
D38	x																		x			

Mark “x” is used to indicate characteristics extracted from definitions.

**Table A3:** Definitions analysis: What actions IUI perform and what do they adapt to.

	Action												Adaptation															
	Adaptation	Personaliation	Learning	Knowledge Obtainment	Customization	Reasoning	Communication	Assistance	Analyzing	Problem-Solving	Modeling	Representing	Context	Subject/User	Users Needs	Users Intent, Goals	Human Behaviour	Gesture	Media	Attention	Discourse	Patterns of Behavior	Domain	Scenario	Environment	Situation	Device	Task
D1	x												x															
D2																												
D3	x		x											x														
D4						x					x	x	x	x													x	x
D5	x																x											
D6	x													x												x	x	
D7																												
D8																												
D9																												
D10	x																					x				x		
D11	x													x														
D12	x																											
D13																				x								
D14		x													x													
D15						x						x		x					x		x		x					x
D16						x				x		x																
D17					x																							
D18																x												
D19						x						x		x					x		x		x					x
D20																												
D21														x														
D22	x														x													
D23																												
D24	x													x														
D25	x						x	x																				
D26	x												x															
D27	x			x					x					x	x													
D28																												
D29																												
D30														x	x													
D31																												
D32	x																											
D33																										x		
D34	x													x			x											
D35	x																							x				
D36	x															x												
D37								x																				
D38	x	x												x											x			

Mark “x” is used to indicate characteristics extracted from definitions.
