# Supplementary material for: Intelligent User Interfaces and Their Evaluation: A Systematic Mapping Study
Source: Sensors (Basel). 2022 Aug 4;22(15):5830. doi: 10.3390/s22155830 (PMC9370954; doi:10.3390/s22155830)
Supplement: Supplementary file 1 [file sensors-22-05830-s001.zip › sensors-1827540-supplementary.pdf]

Supplementary material: Studies included in the systematic mapping study

General						*Evaluation*			
ID	Study	Year	Solution offered	Research Type	Domain	Intelligent entity	UX	Usability	Method
S1	[1]	2022	Methodology	Evaluation research	Software engineering	Interface	Yes	No	Expert based evaluation, User testing
S2	[2]	2022	Evaluation	Validation research	Software engineering	Component	No	No	
S3	[3]	2022	Approach	Validation research	Communication	Interface	No	No	
S4	[4]	2022	Evaluation	Evaluation research	Software engineering	Software engineering	Yes	No	Experiment, Survey
S5	[5]	2022	User study	Validation research	Human-computer interaction	Agent	No	No	
S6	[6]	2022	Model	Validation research	Human-computer interaction	Interaction	No	No	
S7	[7]	2022	Software Solution	Validation research	Human-computer interaction	Other model	No	No	
S8	[8]	2022	Field overview	Literature review	Software engineering	N/A	N/A	N/A	
S9	[9]	2021	Recommender system	Solution proposal	Education	Interface	No	No	
S10	[10]	2021	System	Evaluation research	Software engineering	Interface	Yes	N/A	Focus groups
S11	[11]	2021	Framework	Evaluation research	Software engineering	Interface	No	Yes	User testing, Questionnaire
S12	[12]	2021	HCI Recognition	Solution proposal	Human-computer interaction	Algorithm	No	No	
S13	[13]	2021	User study	Validation research	Human-computer interaction	Agent	Partly	No	
S14	[14]	2021	Framework	Solution proposal	Human-computer interaction	Interface	N/A	N/A	
S15	[15]	2021	Agent	Validation research	Logistics and vehicles	Agent	No	No	
S16	[16]	2021	Model	Validation research	Software engineering	Other model	N/A	N/A	
S17	[17]	2021	Chat bot	Exploratory	Software engineering	Software	No	No	
S18	[18]	2021	User Interface	Evaluation research	Academia	Interface	No	Yes	User testing, Questionnaire
S19	[19]	2021	Framework	Validation research	3D printing	Interface	No	Yes	User testing, Questionnaire
S20	[20]	2021	User Interface	Validation research	Healthcare	Agent	No	No	

General						*Evaluation*			
ID	Study	Year	Solution offered	Research Type	Domain	Intelligent entity	UX	Usability	Method
S21	[21]	2021	User Interface	Validation research	Accessibility	Interface	No	Yes	User Testing
S22	[22]	2021	Recommender system	Solution proposal	Academia	Interface	No	No	
S23	[23]	2021	System	Validation research	Healthcare	System	No	No	
S24	[24]	2021	Approach	Validation research	Healthcare	Other model	N/A	N/A	
S25	[25]	2021	Field overview	Opinion paper	Human-computer interaction	Interface	Partly	No	
S26	[26]	2021	Field overview	Solution proposal	Software engineering	Agent	No	No	
S27	[27]	2021	Pipeline	Validation research	Software engineering	Algorithm	N/A	No	
S28	[28]	2021	Field overview	Literature review	Human-computer interaction	N/A	No*	Yes	
S29	[29]	2020	Approach	Solution proposal	Software engineering	Interface	N/A	N/A	
S30	[30]	2020	Field overview	Solution proposal	Software engineering	N/A	N/A	No	
S31	[31]	2020	Model	Solution proposal	Software engineering	Other model	N/A	N/A	
S32	[32]	2020	Field overview	Literature review	Human-computer interaction	Interaction	N/A	N/A	
S33	[33]	2020	User Interface	Evaluation research	Software engineering	Interface	No*	Yes	User testing, Questionnaire
S34	[34]	2020	Field overview	Literature review	Human-computer interaction	N/A	No	No	
S35	[35]	2020	Interaction	Evaluation research	Accessibility	Interaction	No*	Yes	Questionnaire
S36	[36]	2020	User Interface	Validation research	Other	Interface	No	No	
S37	[37]	2020	framework	Validation research	Human-computer interaction	Other model	N/A	N/A	
S38	[38]	2020	System	Evaluation research	Accessibility	Interface	Partly	No	
S39	[39]	2020	User study	Evaluation research	Human-computer interaction	System	Partly	No	
S40	[40]	2020	User study	Evaluation research	Recommendation	System	Partly	No	
S41	[41]	2020	User study	Evaluation research	Human-computer interaction	Agent	Partly	No	
S42	[42]	2020	User study	Evaluation research	Human-computer interaction	Other model	Yes	No	User testing, Survey
S43	[43]	2020	User Interface	Solution proposal	Communication	Conversational UI	No	No	
S44	[44]	2020	Application	Solution proposal	Healthcare	Application	No	No	

General						*Evaluation*			
ID	Study	Year	Solution offered	Research Type	Domain	Intelligent entity	UX	Usability	Method
S45	[45]	2020	Framework	Validation research	Software engineering	Interface	Yes	No	Automated testing
S46	[46]	2020	User study/insight	Evaluation research	Human-computer interaction	Recommender system	Yes	No	User study, Experiment
S47	[47]	2020	Model	Evaluation research	Human-computer interaction	Method	N/A	N/A	
S48	[48]	2020	User study/insight	Evaluation research	Human-computer interaction	Recommender system	Partly	N/A	
S49	[49]	2020	User Interface	Validation research	Accessibility	Interface	No	No	
S50	[50]	2020	User Interface	Solution proposal	Security	Interface	No	No	
S51	[51]	2020	Agent	Evaluation research	Human-computer interaction	Agent	Yes	No	User testing
S52	[52]	2020	User Interface	Solution proposal	Human-computer interaction	Interface	No	No	
S53	[53]	2019	User Interface	Solution proposal	Logistics and vehicles	Agent	No	No	
S54	[54]	2019	User study	Solution proposal	Human-computer interaction	N/A	N/A	N/A	
S55	[55]	2019	Recommender system	Validation research	Software engineering	Recommender system	No	No	
S56	[56]	2019	Field overview	Literature review	Human-computer interaction	N/A	No	No	
S57	[57]	2019	Process	Solution proposal	Software engineering	Interface	N/A	No	
S58	[58]	2019	Approach	Exploratory	Culture	N/A	N/A	N/A	
S59	[59]	2019	Model	Solution proposal	Human-computer interaction	Other model	N/A	N/A	
S60	[60]	2019	Evaluation	Evaluation research	Logistics and vehicles	Interface	Yes	No	Experiment, Interview
S61	[61]	2019	Evaluation	Evaluation research	Recommendation	Interface	Yes	No	Survey, User testing
S62	[62]	2019	User Interface	Validation research	Security	Interface	Partly	No	
S63	[63]	2019	Technique / Method	Solution proposal	Human-computer interaction	Interface	No	No	
S64	[64]	2019	User Interface	Validation research	Sport	Interface	Partly	No	
S65	[65]	2019	Device	Solution proposal	Human-computer interaction	Machine	No	No	
S66	[66]	2019	Framework	Solution proposal	Software engineering	Interface	N/A	No	
S67	[67]	2019	Application	Evaluation research	Software engineering	Interaction	No	No	

General						*Evaluation*			
ID	Study	Year	Solution offered	Research Type	Domain	Intelligent entity	UX	Usability	Method
S68	[68]	2019	Field overview	Exploratory	Human-computer interaction	Interface	Partly	No	
S69	[69]	2019	User study/insight	Validation research	Recommendation	Recommender system	Partly	No	
S70	[70]	2019	Field overview	Solution proposal	Music	Interface	No	No	
S71	[71]	2019	Agent	Validation research	Insurance	Interface agent	Yes	No	Expert based evaluation, User testing
S72	[72]	2019	Field overview	Evaluation research	Software engineering	Interface	N/A	No	
S73	[73]	2019	User Interface	Validation research	Management and organisation	Interface	Partly	No	
S74	[74]	2019	Recommender system	Evaluation research	Music	Recommender system	Yes	No	User testing
S75	[75]	2019	Agent	Evaluation research	Insurance	Interface agent	Yes	No	User testing
S76	[76]	2019	Technique / Method	Evaluation research	Robotics	Conversational UI	Partly	No	Expert based evaluation, User testing
S77	[77]	2019	Model	Validation research	Education	System	No	No	
S78	[78]	2019	Framework	Solution proposal	Human-computer interaction	System	No	No	
S79	[79]	2018	Framework	Validation research	Software engineering	System	No	No	
S80	[80]	2018	Data collection	Solution proposal	Human-computer interaction	N/A	N/A	N/A	
S81	[81]	2018	System	Solution proposal	Communication	System	No	No	
S82	[82]	2018	Model	Validation research	Sales	Interface	No	No	User study
S83	[83]	2018	Interaction	Validation research	Human-computer interaction	Conversational UI	N/A	N/A	
S84	[84]	2018	User study	Validation research	Accessibility	Interface	No	No	
S85	[85]	2018	User Interface	Evaluation research	Healthcare	Interface	Partly	No	
S86	[86]	2018	User Interface	Validation research	Logistics and vehicles	Conversational UI	No	No	
S87	[87]	2018	System	Validation research	Human-computer interaction	System	N/A	N/A	
S88	[88]	2018	HCI Recognition	Validation research	Entertainment and Games	Software	N/A	N/A	
S87	[89]	2018	Field overview	Literature review	Healthcare	Interface	N/A	N/A	
S89	[90]	2018	User Interface	Validation research	Photography	Interface	Partly	No	Survey

General						*Evaluation*			
ID	Study	Year	Solution offered	Research Type	Domain	Intelligent entity	UX	Usability	Method
S90	[91]	2018	Technique / Method	Validation research	Human-computer interaction	Interface	N/A	No	
S91	[92]	2018	User Interface	Solution proposal	Factory and production	Interface	No	No	
S92	[93]	2018	Browser	Solution proposal	Work and productivity	Interface	No	No	
S93	[94]	2018	Algorithm	Validation research	Finance	Interface	No	No	
S94	[95]	2017	Model	Validation research	Healthcare	Interface	No	No	
S95	[96]	2017	Design space	Solution proposal	Human-computer interaction	Interface	No	No	
S96	[97]	2017	Platform	Validation research	Energy	Interface	No	No	
S97	[98]	2017	Model	Solution proposal	Software engineering	Other model	N/A	N/A	
S98	[99]	2017	User Interface	Validation research	Software engineering	Interface	No	No	
S99	[100]	2017	Approach	Validation research	Healthcare	Algorithm	N/A	N/A	
S100	[101]	2017	User Interface	Validation research	Software engineering	Interface	No	Yes	User testing, Questionnaire, Usability Metrics / Software Metrics
S101	[102]	2017	Evaluation	Evaluation research	Healthcare	Interface	Yes	Yes	Expert based evaluation, "Thinking aloud" Protocol, User testing, Interview
S102	[103]	2017	Technique / Method	Validation research	Software engineering	Recommender system	N/A	N/A	
S103	[104]	2017	Model	Solution proposal	Human-computer interaction	Interface	No	No	
S104	[105]	2017	Prediction	Solution proposal	Human-computer interaction	Interface	No	No	
S105	[106]	2017	User Interface	Solution proposal	Communication	Interface	N/A	N/A	
S106	[107]	2017	Approach	Evaluation research	Statistics and data analytics	Interface	Partly	No	User testing, interview, Survey
S107	[108]	2017	Algorithm	Solution proposal	Health and wellbeing	N/A	N/A	N/A	
S108	[109]	2017	Field overview	Opinion paper	Music	Interface	N/A	N/A	
S109	[110]	2017	System	Solution proposal	Academia	Interface	No	No	
S110	[111]	2017	System	Validation research	Healthcare	Machine	No	No	

General						*Evaluation*			
ID	Study	Year	Solution offered	Research Type	Domain	Intelligent entity	UX	Usability	Method
S111	[112]	2017	User Interface	Solution proposal	Culture	Interface	No	No	
S112	[113]	2017	User Interface	Solution proposal	Human-computer interaction	Interface	No	No	
S113	[114]	2017	Recommender system	Validation research	Fashion	Interface	No	No	
S114	[115]	2017	User Interface	Solution proposal	Other	Interface	No	No	
S115	[116]	2017	Field overview	Opinion paper	Human-computer interaction	Interface	N/A	No	
S116	[117]	2016	Opinion	Solution proposal	Security	N/A	N/A	N/A	
S117	[118]	2016	Methodology	Validation research	Software engineering	System	N/A	N/A	
S118	[119]	2016	Concept	Solution proposal	Software engineering	N/A	N/A	No	
S119	[120]	2016	UI element	Validation research	Human-computer interaction	Component	No	No	
S120	[121]	2016	Application	Solution proposal	Healthcare	Interface	No	No	
S121	[122]	2016	Model	Solution proposal	Software engineering	Other model	N/A	N/A	
S122	[123]	2016	Technique / Method	Validation research	Human-computer interaction	N/A	N/A	N/A	
S123	[124]	2016	Interaction	Solution proposal	Human-computer interaction	Machine	N/A	N/A	
S124	[125]	2016	Field overview	Solution proposal	Cartography and Geolocation	Interface	N/A	N/A	
S125	[126]	2016	User Interface	Validation research	Healthcare	Interface	No	No	
S126	[127]	2016	User study	Validation research	Human-computer interaction	Interface	No	No	
S127	[128]	2016	Algorithm	Validation research	Software engineering	Algorithm	N/A	N/A	
S128	[129]	2016	User Interface	Solution proposal	Software engineering	Interface	N/A	N/A	
S129	[130]	2016	Algorithm	Validation research	Software engineering	Algorithm	N/A	N/A	
S130	[131]	2016	Agent	Evaluation research	Academia	Agent	Yes	No	"Thinking aloud" method, Experiment, User testing
S131	[132]	2016	User Interface	Validation research	Logistics and vehicles	Interface	No	No	
S132	[133]	2016	Agent	Solution proposal	Software engineering	Agent	N/A	N/A	
S133	[134]	2016	Model	Validation research	Software engineering	Interface	No	No	

General						*Evaluation*			
ID	Study	Year	Solution offered	Research Type	Domain	Intelligent entity	UX	Usability	Method
S134	[135]	2016	Technique / Method	Validation research	Software engineering	Algorithm	N/A	N/A	
S135	[136]	2016	Agent	Validation research	Communication	Agent	No	No	
S136	[137]	2016	System	Evaluation research	Healthcare	Interface	Yes	No	Survey, Experiment, Expert based evaluation
S137	[138]	2016	Application	Solution proposal	Health and wellbeing	Interface	No	No	
S138	[139]	2015	Evaluation	Evaluation research	Software engineering	N/A	No*	Yes	User testing
S139	[140]	2015	Assistance	Validation research	Software engineering	Assistance	N/A	N/A	
S140	[141]	2015	Dataset	Validation research	Human-computer interaction	Interface	No	No	
S141	[142]	2015	User study	Validation research	Human-computer interaction	Other model	N/A	N/A	
S142	[143]	2015	Tool	Solution proposal	Statistics and data analytics	Interface	No	No	
S143	[144]	2015	Framework	Validation research	Human-computer interaction	N/A	N/A	N/A	
S144	[145]	2015	User study	Exploratory	Human-computer interaction	N/A	N/A	N/A	
S145	[146]	2015	Approach	Evaluation research	Robotics	Interface	Partly	No	
S146	[147]	2015	User Interface	Validation research	Work and productivity	Interface	No	No	
S147	[148]	2015	Opinion	Opinion paper	Entertainment and Games	Interface	No	No	
S148	[149]	2015	Model	Solution proposal	Software engineering	Interface	No	No	
S149	[150]	2015	Evaluation	Evaluation research	Software engineering	Interface	No	No	
S150	[151]	2015	Framework	Solution proposal	Software engineering	System	No	No	
S151	[152]	2015	Evaluation	Evaluation research	Recommendation	Interface	Yes	Yes	Experiment, User testing, Survey
S152	[153]	2015	Technique / Method	Validation research	Other	Algorithm	N/A	N/A	
S153	[154]	2015	User Interface	Solution proposal	Industry	Interface	No	No	
S154	[155]	2015	Application	Evaluation research	Education	Application	Partly	No	User testing
S155	[156]	2015	Methodology	Solution proposal	Software engineering	Interface	No	No	
S156	[157]	2015	Technique / Method	Solution proposal	Human-computer interaction	Interface	No	No	

General						*Evaluation*			
ID	Study	Year	Solution offered	Research Type	Domain	Intelligent entity	UX	Usability	Method
S157	[158]	2015	Methodology	Validation research	Human-computer interaction	Interface	No*	Yes	
S158	[159]	2015	User Interface	Evaluation research	Human-computer interaction	Interface	Partly	No	User testing, Survey
S159	[160]	2015	Ontology	Solution proposal	Robotics	Interface	No	No	
S160	[161]	2015	Platform	Validation research	Recommendation	Interface	No	No	
S161	[162]	2015	System	Validation research	Software engineering	System	N/A	N/A	
S162	[163]	2014	Technique / Method	Evaluation research	Security	Component	No*	Yes	User Testing, Usability Metrics / Software Metrics
S163	[164]	2014	System	Validation research	Software engineering	System	No	No	
S164	[165]	2014	Model	Solution proposal	Accessibility	User model	No	No	
S165	[166]	2014	Approach	Validation research	Security	Interface	No	No	
S166	[167]	2014	System	Solution proposal	Software engineering	Algorithm	N/A	N/A	
S167	[168]	2014	System	Validation research	Logistics and vehicles	Interface	No	No	
S168	[169]	2014	User Interface	Validation research	Human-computer interaction	Interface	No	No	
S169	[170]	2014	User Interface	Solution proposal	Statistics and data analytics	Interface	No	No	
S170	[171]	2014	User Interface	Solution proposal	Logistics and vehicles	Interface	No	No	
S171	[172]	2014	Process	Solution proposal	Software engineering	Interface	No	No	
S172	[173]	2014	Characteristics analysis	Solution proposal	Software engineering	Interface	No	No	
S173	[174]	2014	Model	Validation research	Software engineering	Interface	No*	Partly	User testing
S174	[175]	2014	Tool	Validation research	Recommendation	Recommender system	No	No	
S175	[176]	2014	Technique / Method	Solution proposal	Recommendation	Recommender system	No	No	
S176	[177]	2014	System	Validation research	Music	Interface	Partly	No	User testing
S177	[178]	2014	User Interface	Validation research	Healthcare	Dialogue system	No	No	
S178	[179]	2014	Evaluation	Evaluation research	Healthcare	Interface	Yes	Yes	User testing, Survey
S179	[180]	2014	UI element	Validation research	Human-computer interaction	Interface	No	No	
S180	[181]	2014	Tool	Evaluation research	Audio	Interface	Yes	No	User testing, Survey

General						*Evaluation*			
ID	Study	Year	Solution offered	Research Type	Domain	Intelligent entity	UX	Usability	Method
S181	[182]	2014	Agent	Validation research	Communication	Agent	Partly	No	User testing
S182	[183]	2013	Model	Solution proposal	Software engineering	Other model	N/A	N/A	
S183	[184]	2013	Algorithm	Validation research	Software engineering	Method	N/A	N/A	
S184	[185]	2013	Ontology	Solution proposal	Software engineering	Interface	No	N/A	
S185	[186]	2013	System	Evaluation research	Human-computer interaction	Interface	Partly	No	Interview with users
S186	[187]	2013	Tool	Validation research	Management and organisation	Interface	No	No	
S187	[188]	2013	Agent	Validation research	Human-computer interaction	Agent	No	No	
S188	[189]	2013	Agent	Evaluation research	Communication	Other model	Partly	No	User testing, Survey
S189	[190]	2013	System	Validation research	Health and wellbeing	System	No	No	
S190	[191]	2013	Agent	Evaluation research	Communication	Agent	Partly	No	User testing, Experiment
S191	[192]	2013	User study/insight	Validation research	Security	Interface	No*	Yes	User testing, Survey
S192	[193]	2013	User Interface	Solution proposal	Software engineering	Interface	No	No	
S193	[194]	2013	System	Evaluation research	Logistics and vehicles	Interface	No*	Yes	User testing, Interview
S194	[195]	2013	Algorithm	Validation research	Communication	Algorithm	N/A	N/A	
S195	[196]	2013	Evaluation	Evaluation research	Human-computer interaction	Dialogue system	Yes	No	Experiment
S196	[197]	2013	User Interface	Solution proposal	Energy	Interface	No	No	
S197	[198]	2013	Tool	Validation research	Software engineering	Interface	No	No	
S198	[199]	2012	Device	Solution proposal	Military	Interface	No	No	
S199	[200]	2012	System	Validation research	Health and wellbeing	Interaction	N/A	N/A	
S200	[201]	2012	Framework	Literature review	Human-computer interaction	N/A	N/A		
S201	[202]	2012	Assistant	Solution proposal	Logistics and vehicles	Interface	N/A	N/A	
S202	[203]	2012	Approach	Validation research	Software engineering	Agent	No	No	
S203	[204]	2012	System	Evaluation research	Human-computer interaction	System	No*	Yes	User testing, Survey
S204	[205]	2012	Technique / Method	Evaluation research	Healthcare	Interface	No*	Yes	User testing
S205	[206]	2012	Architecture	Validation research	Government	Agent	No	No	

General						*Evaluation*			
ID	Study	Year	Solution offered	Research Type	Domain	Intelligent entity	UX	Usability	Method
S206	[207]	2012	Approach	Solution proposal	Software engineering	Agent	No	No	
S207	[208]	2012	Agent	Validation research	Human-computer interaction	Agent	N/A	N/A	
S208	[209]	2012	Plugin	Validation research	Recommendation	Tool	No	No	
S209	[210]	2012	User Interface	Evaluation research	Accessibility	Interface	No*	Yes	User testing
S210	[211]	2011	Technique / Method	Validation research	Software engineering	Interface	No	No	
S212	[212]	2012	Approach	Validation research	Work and productivity	Agent	No	No	

*No: User experience was evaluated indirectly, via usability; UX evaluation was not mentioned in the study. N/A: Not applicable

References

[1] Zinovia Stefanidi, George Margetis, Stavroula Ntoa, and George Papagiannakis. Real-time adaptation of context-aware intelligent user interfaces, for enhanced situational awareness. *IEEE Access*, 10:23367–23393, 2022.

[2] Jianlong Zhou, Xianglin Miao, Feijuan He, and Yalin Miao. Effects of font style and font color in news text on user cognitive load in intelligent user interfaces. *IEEE Access*, 10:10719–10730, 2022.

[3] Jung In Koh, Samantha Ray, Josh Cherian, Paul Taele, and Tracy Hammond. Show of hands: Leveraging hand gestural cues in virtual meetings for intelligent impromptu polling interactions. In *27th International Conference on Intelligent User Interfaces*, IUI '22, page 292–309, New York, NY, USA, 2022. Association for Computing Machinery.

[4] Justin D. Weisz, Michael Muller, Steven I. Ross, Fernando Martinez, Stephanie Houde, Mayank Agarwal, Kartik Talamadupula, and John T. Richards. Better together? an evaluation of ai-supported code translation. In *27th International Conference on Intelligent User Interfaces*, IUI '22, page 369–391, New York, NY, USA, 2022. Association for Computing Machinery.

[5] Reut Asraf, Chen Rozenshtein, and David Sarne. The positive effect of user faults over agent perception in collaborative settings and its use in agent design. In *Distributed Artificial Intelligence: Third International Conference, DAI 2021, Shanghai, China, December 17–18, 2021, Proceedings*, page 129–149, Berlin, Heidelberg, 2021. Springer-Verlag.

[6] Hu wenjuan, R. Premalatha, and R. S. Aiswarya. Physical education system and training framework based on human–computer interaction for augmentative and alternative communication. *International Journal of Speech Technology*, 25(2):367–377, Jun 2022.

[7] Holly Burrows, Javad Zarrin, Lakshmi Babu-Saheer, and Mahdi Maktab-Dar-Oghaz. Realtime emotional reflective user interface based on deep convolutional neural networks and generative adversarial networks. *Electronics*, 11(1), 2022.

[8] Boštjan Šumak, Saša Brdnik, and Maja Pušnik. Sensors and artificial intelligence methods and algorithms for human&computer intelligent interaction: A systematic mapping study. *Sensors*, 22(1), 2022.

[9] Behnam Rahdari, Peter Brusilovsky, and Alireza Javadian Sabet. *Connecting Students with Research Advisors Through User-Controlled Recommendation*, page 745–748. Association for Computing Machinery, New York, NY, USA, 2021.

[10] Matthew Runyon, Seth Polsley, Blake Williford, Sin-Ning Cindy Liu, Josh Hurt, Julie Linsey, and Tracy Hammond. An intelligent system to analyze sketched solutions to open-ended truss problems. In *26th International Conference on Intelligent User Interfaces*, IUI '21, page 224–233, New York, NY, USA, 2021. Association for Computing Machinery.

[11] Yolanda Gil, Daniel Garijo, Deborah Khider, Craig A. Knoblock, Varun Ratnakar, Maximiliano Osorio, Hernán Vargas, Minh Pham, Jay Pujara, Basel Shbita, Binh Vu, Yao-Yi Chiang, Dan Feldman, Yijun Lin, Hayley Song, Vipin Kumar, Ankush Khandelwal, Michael Steinbach, Kshitij Tayal, Shaoming Xu, Suzanne A. Pierce, Lissa Pearson, Daniel Hardesty-Lewis, Ewa Deelman, Rafael Ferreira Da Silva, Rajiv Mayani, Armen R. Kemanian, Yuning Shi, Lorne Leonard, Scott Peckham, Maria Stoica, Kelly Cobourn, Zeya Zhang, Christopher Duffy, and Lele Shu. Artificial intelligence for modeling complex systems: Taming the complexity of expert models to improve decision making. *ACM Trans. Interact. Intell. Syst.*, 11(2), jul 2021.

[12] Radoslaw Niewiadomski and Alessandra Sciutti. Multimodal emotion recognition of hand-object interaction. In *26th International Conference on Intelligent User Interfaces*, IUI '21, page 351–355, New York, NY, USA, 2021. Association for Computing Machinery.

[13] Reut Asraf, Chen Rozenshtein, and David Sarne. On the effect of user faults on her perception of agent's faults in collaborative settings. HAI '21, page 372–376, New York, NY, USA, 2021. Association for Computing Machinery.

[14] Silvia Abrahão, Emilio Insfran, Arthur Sluÿters, and Jean Vanderdonckt. Model-based intelligent user interface adaptation: challenges and future directions. *Software and Systems Modeling*, 20(5):1335–1349, Oct 2021.

[15] Jakob Kuen, Clemens Schartmüller, and Philipp Wintersberger. *The TOR Agent:Optimizing Driver Take-Over with Reinforcement Learning*, page 47–52. Association for Computing Machinery, New York, NY, USA, 2021.

[16] Robert E. Bixler and Sidney K. D'Mello. Crossed eyes: Domain adaptation for gaze-based mind wandering models. In *ACM Symposium on Eye Tracking Research and Applications*, ETRA '21 Full Papers, New York, NY, USA, 2021. Association for Computing Machinery.

[17] Prasanth Murali, Javier Hernandez, Daniel McDuff, Kael Rowan, Jina Suh, and Mary Czerwinski. Affectivespotlight: Facilitating the communication of affective responses from audience members during online presentations. In *Proceedings of the 2021 CHI Conference on Human Factors in Computing Systems*, CHI '21, New York, NY, USA, 2021. Association for Computing Machinery.

[18] Allard Oelen, Markus Stocker, and Sören Auer. Crowdsourcing scholarly discourse annotations. In *26th International Conference on Intelligent User Interfaces*, IUI '21, page 464–474, New York, NY, USA, 2021. Association for Computing Machinery.

[19] Alexander Berman, Ketan Thakare, Joshua Howell, Francis Quek, and Jeeeun Kim. Howdiy: Towards meta-design tools to support anyone to 3d print anywhere. In *26th International Conference on Intelligent User Interfaces*, IUI '21, page 491–503, New York, NY, USA, 2021. Association for Computing Machinery.

[20] Mohamed Hachem Kermani, Zizette Boufaïda, Sabrina Benredjem, and Amani Saker. An mvc-inspired approach for an intelligent annotation of a protein ontology : Ia-pronto. 13:308–318, 10 2021.

[21] Varun John Prajakta Thakur, Tanay Dalvi and Swati Chandna. Magvi: Towards saliency-driven video magnification application for the people with low vision. ACM, April 2020.

[22] Behnam Rahdari, Peter Brusilovsky, and Alireza Javadian Sabet. Controlling personalized recommendations in two dimensions with a carousel-based interface. In *IntRS@RecSys*, 2021.

[23] Syed Hammad Hussain Shah, Bjørnar Longva, Ibrahim A. Hameed, Mads Solberg, and Anniken Susanne T. Karlsen. Health data management for nursing practice: An intelligent, holographic mixed-reality system. In Constantine Stephanidis, Margherita Antona, and Stavroula Ntoa, editors, *HCI International 2021 - Posters*, pages 329–336, Cham, 2021. Springer International Publishing.

[24] Projna Paromita, Theodora Chaspari, Seyed Sajjadi, Anurag Das, B. Mortazavi, and Ricardo Gutierrez-Osuna. Personalized meal classification using continuous glucose monitors. In *IUI Workshops*, 2021.

[25] Timothy I. Michaels, Sonali Singal, Emily Stone, Laura Braider, and John Kane. Digital tools for the treatment of borderline personality disorder: A critical review of smartphone applications. In *IUI Workshops*, 2021.

[26] Xinyu Huang, Fridolin Wild, and Denise Whitelock. Design dimensions for holographic intelligent agents: A comparative analysis. In *1st International Workshop on Multimodal Artificial Intelligence in- Education, MAIED 2021*, 2021.

[27] Yu Zhang, Bob Coecke, and Min Chen. Mi3: Machine-initiated intelligent interaction for interactive classification and data reconstruction. *ACM Trans. Interact. Intell. Syst.*, 11(3–4), aug 2021.

[28] Mahdi H. Miraz, Maaruf Ali, and Peter S. Excell. Adaptive user interfaces and universal usability through plasticity of user interface design. *Computer Science Review*, 40:100363, 2021.

[29] Andriy Pavlov, Halyna Pidnebesna, and Volodymyr Stepashko. Ontology-based approach to construction of intelligent interface for inductive modeling tools. In *2020 IEEE 15th International Conference on Computer Sciences and Information Technologies (CSIT)*, volume 2, pages 26–29, 2020.

[30] Abdallah Namoun, Abdullah Alshanqiti, Ezzat Chamudi, and Mohammed Ayman Rahmon. Web design scraping: Enabling factors, opportunities and research directions. In *2020 12th International Conference on Information Technology and Electrical Engineering (ICITEE)*, pages 104–109, 2020.

[31] Ben Khayut, Lina Fabri, and Maya Avikhana. Toward general ai: Consciousness computational modeling under uncertainty. In *2020 International Conference on Mathematics and Computers in Science and Engineering (MACISE)*, pages 90–97, 2020.

[32] Alex Shaw, Jaime Ruiz, and Lisa Anthony. A Survey on Applying Automated Recognition of Touchscreen Stroke Gestures to Children's Input. *Interacting with Computers*, 32(5-6):524–547, 04 2021.

[33] Dmytro Zhelezniakov, Anastasiia Cherneha, Viktor Zaytsev, Tetiana Ignatova, Olga Radyvonenko, and Oleg Yakovchuk. Evaluating new requirements to pen-centric intelligent user interface based on end-to-end mathematical expressions recognition. In *Proceedings of the 25th International Conference on Intelligent User Interfaces*, IUI '20, page 212–220, New York, NY, USA, 2020. Association for Computing Machinery.

[34] Sarah Theres Völkel, Christina Schneegass, Malin Eiband, and Daniel Buschek. What is "intelligent" in intelligent user interfaces? a meta-analysis of 25 years of iui. In *Proceedings of the 25th International Conference on Intelligent User Interfaces*, IUI '20, page 477–487, New York, NY, USA, 2020. Association for Computing Machinery.

[35] Rúbia E. O. Schultz Ascari, Luciano Silva, and Roberto Pereira. Personalized gestural interaction applied in a gesture interactive game-based approach for people with disabilities. In *Proceedings of the 25th International Conference on Intelligent User Interfaces*, IUI '20, page 100–110, New York, NY, USA, 2020. Association for Computing Machinery.

[36] Blake Williford, Matthew Runyon, and Tracy Hammond. Recognizing perspective accuracy: An intelligent user interface for assisting novices. In *Proceedings of the 25th International Conference on Intelligent User Interfaces*, IUI '20, page 231–242, New York, NY, USA, 2020. Association for Computing Machinery.

[37] Runze Gan, Jiaming Liang, Bashar I. Ahmad, and Simon Godsill. Modeling intent and destination prediction within a bayesian framework: Predictive touch as a usecase. *Data-Centric Engineering*, 1:e12, 2020.

[38] Angela Constantinescu, Karin Müller, Monica Haurilet, Vanessa Petrausch, and Rainer Stiefelhagen. *Bring the Environment to Life: A Sonification Module for People with Visual Impairments to Improve Situation Awareness*, page 50–59. Association for Computing Machinery, New York, NY, USA, 2020.

[39] Wei Guo, Byeong-Young Cho, and Jingtao Wang. *StrategicReading: Understanding Complex Mobile Reading Strategies via Implicit Behavior Sensing*, page 491–500. Association for Computing Machinery, New York, NY, USA, 2020.

[40] Fabio Colella, Pedram Daee, Jussi Jokinen, Antti Oulasvirta, and Samuel Kaski. *Human Strategic Steering Improves Performance of Interactive Optimization*, page 293–297. Association for Computing Machinery, New York, NY, USA, 2020.

[41] Andrew Anderson, Jonathan Dodge, Amrita Sadarangani, Zoe Juozapaitis, Evan Newman, Jed Irvine, Souti Chattopadhyay, Matthew Olson, Alan Fern, and Margaret Burnett. Mental models of mere mortals with explanations of reinforcement learning. *ACM Trans. Interact. Intell. Syst.*, 10(2), may 2020.

[42] Alison Smith-Renner, Varun Kumar, Jordan Boyd-Graber, Kevin Seppi, and Leah Findlater. Digging into user control: Perceptions of adherence and instability in transparent models. In *Proceedings of the 25th International Conference on Intelligent User Interfaces*, IUI '20, page 519–530, New York, NY, USA, 2020. Association for Computing Machinery.

[43] Abhishek Kaushik, Vishal Bhat Ramachandra, and Gareth J. F. Jones. *An Interface for Agent Supported Conversational Search*, page 452–456. Association for Computing Machinery, New York, NY, USA, 2020.

[44] Catherine Pagiatakis, David Rivest-Hénault, David Roy, Francis Thibault, and Di Jiang. Intelligent interaction interface for medical emergencies: Application to mobile hypoglycemia management. *Smart Health*, 15:100091, 2020.

[45] Vivien Johnston, Michaela M. Black, and Jonathan G. Wallace. A holistic ux methodological framework for measuring the aspects of how dynamic, adaptive and intelligent a software solution is and make recommendations for improvement. 2020.

[46] Pigi Kouki, James Schaffer, Jay Pujara, John O'Donovan, and Lise Getoor. Generating and understanding personalized explanations in hybrid recommender systems. *ACM Trans. Interact. Intell. Syst.*, 10(4), nov 2020.

[47] Bing Liu, Ping Fu, Tingwei Chen, Yongqiang Li, and Jeng-Shyang Pan. Facial landmarks detection under occlusions via extended restricted boltzmann machine. *Journal of Internet Technology*, 21(5):1233 – 1241, 2020. Cited by: 1.

[48] Chun-Hua Tsai and Peter Brusilovsky. User feedback in controllable and explainable social recommender systems: A linguistic analysis. volume 2682, page 1 – 13, 2020. Cited by: 1.

[49] Ildar Kagirov, Dmitry Ryumin, and Miloš Železný. Gesture-based intelligent user interface for control of an assistive mobile information robot. In *Interactive Collaborative Robotics: 5th International Conference, ICR 2020, St Petersburg, Russia, October 7-9, 2020, Proceedings*, page 126–134, Berlin, Heidelberg, 2020. Springer-Verlag.

[50] Joseph Aneke, Carmelo Ardito, and Giuseppe Desolda. Designing an intelligent user interface for preventing phishing attacks. In *Beyond Interactions: INTERACT 2019 IFIP TC 13 Workshops, Paphos, Cyprus, September 2–6, 2019, Revised Selected Papers*, page 97–106, Berlin, Heidelberg, 2019. Springer-Verlag.

[51] Dolça Tellols, Maite López-Sánchez, Inmaculada Rodríguez, Pablo Almajano, and Anna Puig. Enhancing sentient embodied conversational agents with machine learning. *Pattern Recognit. Lett.*, 129:317–323, 2020.

[52] Ildar Kagirov, Alexey Karpov, Irina S. Kipyatkova, Konstantin Klyuzhev, Alexander Kudryavcev, Igor Kudryavcev, and Dmitry Ryumin. Lower limbs exoskeleton control system based on intelligent human-machine interface. In *IDC*, 2019.

[53] Hangli Ge, Takeo Hamada, Takahiro Sumitomo, and Noboru Koshizuka. Intellevator: Enhancing elevator system efficiency by proactive computing on the traffic flow. In *2019 IEEE 1st Global Conference on Life Sciences and Technologies (LifeTech)*, pages 80–84, 2019.

[54] Graça Trindade and Raul M. S. Laureano. Profiling the two most populous generations of the piigs countries in the workplace based in online consumption of cultural contents. In *2019 14th Iberian Conference on Information Systems and Technologies (CISTI)*, pages 1–6, 2019.

[55] Shudan Zhong and Hong Xu. Intelligently recommending key bindings on physical keyboards with demonstrations in emacs. In *Proceedings of the 24th International Conference on Intelligent User Interfaces*, IUI '19, page 12–17, New York, NY, USA, 2019. Association for Computing Machinery.

[56] Taisa G. Gonçalves, Christophe Kolski, Káthia M. de Oliveira, Guilherme H. Travassos, and Emmanuelle Grislin-Le Strugeon. A systematic literature review on intelligent user interfaces: Preliminary results. In *Proceedings of the 31st Conference on l'Interaction Homme-Machine: Adjunct*, IHM '19, New York, NY, USA, 2019. Association for Computing Machinery.

[57] Taísa Gonçalves and Ana Rocha. Development process for intelligent user interfaces: an initial approach. In *SBQS'19: Proceedings of the XVIII Brazilian Symposium on Software Quality*, pages 210–215, 10 2019.

[58] Moayad Mokatren, Veronika Bogina, Alan Wecker, and Tsvi Kuflik. A museum visitors classification based on behavioral and demographic features. In *Adjunct Publication of the 27th Conference on User Modeling, Adaptation and Personalization*, UMAP'19 Adjunct, page 383–386, New York, NY, USA, 2019. Association for Computing Machinery.

[59] Jun Wang, Eugene Yujun Fu, Grace Ngai, Hong Va Leong, and Michael Xuelin Huang. Detecting stress from mouse-gaze attraction. In *Proceedings of the 34th ACM/SIGAPP Symposium on Applied Computing*, SAC '19, page 692–700, New York, NY, USA, 2019. Association for Computing Machinery.

[60] Anna-Katharina Frison, Philipp Wintersberger, Tianjia Liu, and Andreas Riener. Why do you like to drive automated? a context-dependent analysis of highly automated driving to elaborate requirements for intelligent user interfaces. In *Proceedings of the 24th International Conference on Intelligent User Interfaces*, IUI '19, page 528–537, New York, NY, USA, 2019. Association for Computing Machinery.

[61] Chun-Hua Tsai and Peter Brusilovsky. Explaining recommendations in an interactive hybrid social recommender. In *Proceedings of the 24th International Conference on Intelligent User Interfaces*, IUI '19, page 391–396, New York, NY, USA, 2019. Association for Computing Machinery.

[62] Tianyi Li, Gregorio Convertino, Ranjeet Kumar Tayi, and Shima Kazerooni. What data should i protect? recommender and planning support for data security analysts. In *Proceedings of the 24th International Conference on Intelligent User Interfaces*, IUI '19, page 286–297, New York, NY, USA, 2019. Association for Computing Machinery.

[63] E. Chae, J. Lee, J. Hwang, and H. Pak. The intelligent user interface system based on 3d digital actor and utilization. In *Journal of Theoretical and Applied Information Technology*, 2019.

[64] Federico Maria Cau, Mattia Samuel Mancosu, Fabrizio Mulas, Paolo Pilloni, and Lucio Davide Spano. An intelligent interface for supporting coaches in providing running feedback. In *Proceedings of the 13th Biannual Conference of the Italian SIGCHI Chapter: Designing the next Interaction*, CHItaly '19, New York, NY, USA, 2019. Association for Computing Machinery.

[65] Heinrich Ruser, André Kaltenbach, and Lars Mechold. *"SmartPointer": Buttonless Remote Control Based on Structured Light and Intuitive Gestures.* Association for Computing Machinery, New York, NY, USA, 2019.

[66] Vivien Johnston, Michaela Black, Jonathan Wallace, Maurice Mulvenna, and Raymond Bond. A framework for the development of a dynamic adaptive intelligent user interface to enhance the user experience. In *Proceedings of the 31st European Conference on Cognitive Ergonomics*, ECCE 2019, page 32–35, New York, NY, USA, 2019. Association for Computing Machinery.

[67] Fanglin Chen and Jason I. Hong. Personal bits: Mining interaction traces for personalized task intelligence. In *Adjunct Proceedings of the 2019 ACM International Joint Conference on Pervasive and Ubiquitous Computing and Proceedings of the 2019 ACM International Symposium on Wearable Computers*, UbiComp/ISWC '19 Adjunct, page 358–362, New York, NY, USA, 2019. Association for Computing Machinery.

[68] Jean Vanderdonckt, Sara Bouzit, Gaëlle Calvary, and Denis Chêne. Exploring a design space of graphical adaptive menus: Normal vs. small screens. *ACM Trans. Interact. Intell. Syst.*, 10(1), jul 2019.

[69] Thi Ngoc Trang Tran, Alexander Felfernig, Viet Man Le, Müslüm Atas, Martin Stettinger, and Ralph Samer. User interfaces for counteracting decision manipulation in group recommender systems. In *Adjunct Publication of the 27th Conference on User Modeling, Adaptation and Personalization*, UMAP'19 Adjunct, page 93–98, New York, NY, USA, 2019. Association for Computing Machinery.

[70] Peter Knees, Markus Schedl, and Masataka Goto. Intelligent user interfaces for music discovery: The past 20 years and what's to come. In *ISMIR*, 2019.

[71] Falko Koetter, Matthias Blohm, Jens Drawehn, Monika Kochanowski, Joscha Goetzer, Daniel Graziotin, and Stefan Wagner. Conversational agents for insurance companies: From theory to practice. In *Agents and Artificial Intelligence: 11th International Conference, ICAART 2019, Prague, Czech Republic, February 19–21, 2019, Revised Selected Papers*, page 338–362, Berlin, Heidelberg, 2019. Springer-Verlag.

[72] Lucio Davide Spano. Understanding each-other: Engineering challenges and opportunities for users and systems in the deep learning era. volume 2503, page 56 – 62, 2019. Cited by: 0.

[73] Lars Schütz. and Korinna Bade. Assessment user interface: Supporting the decision-making process in participatory processes. In *Proceedings of the 21st International Conference on Enterprise Information Systems - Volume 2: ICEIS,*, pages 398–409. INSTICC, SciTePress, 2019.

[74] Pigi Kouki, James Schaffer, Jay Pujara, John O'Donovan, and Lise Getoor. Personalized explanations for hybrid recommender systems. In *Proceedings of the 24th International Conference on Intelligent User Interfaces*, IUI '19, page 379–390, New York, NY, USA, 2019. Association for Computing Machinery.

[75] Falko Koetter, Matthias Blohm, Monika Kochanowski, Joscha Goetzer, Daniel Graziotin, and Stefan Wagner. Motivations, classification and model trial of conversational agents for insurance companies. pages 19–30, 01 2019.

[76] Zeynep Dogmus, Esra Erdem, and Volkan Patoglu. Rehabrobo-query: Answering natural language queries about rehabilitation robotics ontology on the cloud. *Semantic Web*, 10:605–629, 2019.

[77] Alok Baikadi, Lee Becker, Jill Budden, Peter W. Foltz, Andrew Gorman, Scott Hellman, William Murray, and Mark Rosenstein. An apprenticeship model for human and ai collaborative essay grading. In *IUI Workshops*, 2019.

[78] Simone Stumpf. Horses for courses: Making the case for persuasive engagement in smart systems. In *IUI Workshops*, 2019.

[79] Hua-Zhe Tan, Wei Zhao, and Hai-Hua Shen. Adaptive user interface optimization for multi-screen based on machine learning. In *2018 IEEE 22nd International Conference on Computer Supported Cooperative Work in Design ((CSCWD))*, pages 743–748, 2018.

[80] Laurence Devillers, Sophie Rosset, Guillaume Dubuisson Duplessis, Lucile Bechade, Yucel Yemez, Bekir B. Turker, Metin Sezgin, Engin Erzin, Kevin El Haddad, Stephane Dupont, Paul Deleglise, Yannick Esteve, Carole Lailler, Emer Gilmartin, and Nick Campbell. Multifaceted engagement in social interaction with a machine: The joker project. In *2018 13th IEEE International Conference on Automatic Face & Gesture Recognition (FG 2018)*, pages 697–701, 2018.

[81] Phuong Le-Hong and Duc-Thien Bui. A factoid question answering system for vietnamese. In *Companion Proceedings of the The Web Conference 2018*, WWW '18, page 1049–1055, Republic and Canton of Geneva, CHE, 2018. International World Wide Web Conferences Steering Committee.

[82] Oznur Alkan, Elizabeth M. Daly, and Inge Vejsbjerg. Opportunity team builder for sales teams. In *23rd International Conference on Intelligent User Interfaces*, IUI '18, page 251–261, New York, NY, USA, 2018. Association for Computing Machinery.

[83] Diego Gonzalez and Andrew S. Gordon. Comparing speech and text input in interactive narratives. In *23rd International Conference on Intelligent User Interfaces*, IUI '18, page 141–145, New York, NY, USA, 2018. Association for Computing Machinery.

[84] Julia Woodward, Zari McFadden, Nicole Shiver, Amir Ben-hayon, Jason C. Yip, and Lisa Anthony. Using co-design to examine how children conceptualize intelligent interfaces. In *Proceedings of the 2018 CHI Conference on Human Factors in Computing Systems*, CHI '18, page 1–14, New York, NY, USA, 2018. Association for Computing Machinery.

[85] Ivania Donoso-Guzmán and Denis Parra. An interactive relevance feedback interface for evidence-based health care. In *23rd International Conference on Intelligent User Interfaces*, IUI '18, page 103–114, New York, NY, USA, 2018. Association for Computing Machinery.

[86] Peter A. M. Ruijten, Jacques M. B. Terken, and Sanjeev N. Chandramouli. Enhancing trust in autonomous vehicles through intelligent user interfaces that mimic human behavior. *Multimodal Technologies and Interaction*, 2(4), 2018.

[87] Wei Guo and Jingtao Wang. Understanding mobile reading via camera based gaze tracking and kinematic touch modeling. In *Proceedings of the 20th ACM International Conference on Multimodal Interaction*, ICMI '18, page 288–297, New York, NY, USA, 2018. Association for Computing Machinery.

[88] Joshua Newn. Enabling intent recognition through gaze awareness in user interfaces. In *Extended Abstracts of the 2018 CHI Conference on Human Factors in Computing Systems*, CHI EA '18, page 1–4, New York, NY, USA, 2018. Association for Computing Machinery.

[89] Cristina Sanchez, Priscila Cedillo, and Alexandra Bermeo. A systematic mapping study for intelligent user interfaces - iui. In *2017 International Conference on Information Systems and Computer Science (INCISCOS)*, pages 361–368, 2017.

[90] Saif Alabachi and Gita Reese Sukthankar. Intelligently assisting human-guided quadcopter photography. *ArXiv*, abs/1806.08039, 2018.

[91] Sven Stauden, Michael Barz, and Daniel Sonntag. Visual search target inference using bag of deep visual words. In Frank Trollmann and Anni-Yasmin Turhan, editors, *KI 2018: Advances in Artificial Intelligence*, pages 297–304, Cham, 2018. Springer International Publishing.

[92] Mario Heinz, Sebastian Büttner, Martin Wegerich, Frank Marek, and Carsten Röcker. A multi-level localization system for intelligent user interfaces. In Norbert Streitz and Shin'ichi Konomi, editors, *Distributed, Ambient and Pervasive Interactions: Technologies and Contexts*, pages 38–47, Cham, 2018. Springer International Publishing.

[93] Ali Khalili, Peter van den Besselaar, and Klaas Andries de Graaf. Ferasat: A serendipity-fostering faceted browser for linked data. In Aldo Gangemi, Roberto Navigli, Maria-Esther Vidal, Pascal Hitzler, Raphaël Troncy, Laura Hollink, Anna Tordai, and Mehwish Alam, editors, *The Semantic Web*, pages 351–366, Cham, 2018. Springer International Publishing.

[94] Igor Podgorny and Chris Gielow. Semi-automated prevention and curation of duplicate content in social support systems. In *Joint Proceedings of the ACM IUI 2018 Workshops co-located with the 23rd ACM Conference on Intelligent User Interfaces (ACM IUI 2018), Tokyo, Japan, March 11, 2018*, 03 2018.

[95] A. M. Syskov, V. I. Borisov, and V. S. Kublanov. Intelligent multimodal user interface for telemedicine application. In *2017 25th Telecommunication Forum (TELFOR)*, pages 1–4, 2017.

[96] Sarah Bouzit, Gaëlle Calvary, Joëlle Coutaz, Denis Chêne, Eric Petit, and Jean Vanderdonckt. The pda-lpa design space for user interface adaptation. In *2017 11th International Conference on Research Challenges in Information Science (RCIS)*, pages 353–364, 2017.

[97] Lucas Pereira, Miguel Ribeiro, and Nuno Nunes. Engineering and deploying a hardware and software platform to collect and label non-intrusive load monitoring datasets. In *2017 Sustainable Internet and ICT for Sustainability (SustainIT)*, pages 1–9, 2017.

[98] Ben Khayut, Lina Fabri, and Maya Avikhana. Modeling of computational perception of reality, situational awareness, cognition and machine learning under uncertainty. In *2017 Intelligent Systems Conference (IntelliSys)*, pages 331–340, 2017.

[99] Georgios Theocharous, Nikos Vlassis, and Zheng Wen. An interactive points of interest guidance system. IUI '17 Companion, page 49–52, New York, NY, USA, 2017. Association for Computing Machinery.

[100] Shahram Eivazi, Michael Slupina, Wolfgang Fuhl, Hoorieh Afkari, Ahmad Hafez, and Enkelejda Kasneci. Towards automatic skill evaluation in microsurgery. In *Proceedings of the 22nd International Conference on Intelligent User Interfaces Companion*, IUI '17 Companion, page 73–76, New York, NY, USA, 2017. Association for Computing Machinery.

[101] Tavita Su'a, Sherlock A. Licorish, Bastin Tony Roy Savarimuthu, and Tobias Langlotz. Quickreview: A novel data-driven mobile user interface for reporting problematic app features. In *Proceedings of the 22nd International Conference on Intelligent User Interfaces*, IUI '17, page 517–522, New York, NY, USA, 2017. Association for Computing Machinery.

[102] Fatih Demir, Salman Ahmad, Prasad Calyam, Duo Jiang, Rui Huang, and Isa Jahnke. A next-generation augmented reality platform for mass casualty incidents (mci). *J. Usability Studies*, 12(4):193–214, aug 2017.

[103] Saikishore Kalloori. Pairwise preferences and recommender systems. In *Proceedings of the 22nd International Conference on Intelligent User Interfaces Companion*, IUI '17 Companion, page 169–172, New York, NY, USA, 2017. Association for Computing Machinery.

[104] Landy Rajaonarivo, Matthieu Courgeon, Eric Maisel, and Pierre De Loor. Inline co-evolution between users and information presentation for data exploration. In *Proceedings of the 22nd International Conference on Intelligent User Interfaces*, IUI '17, page 215–219, New York, NY, USA, 2017. Association for Computing Machinery.

[105] Anne-Marie Brouwer, Jasper S. van der Waa, Maarten A. Hogervorst, Alessia Cacace, and Hans Stokking. A feasible bci in real life: Using predicted head rotation to improve hmd imaging. In *Proceedings of the 2017 ACM Workshop on An Application-Oriented Approach to BCI out of the Laboratory*, BCIforReal '17, page 35–38, New York, NY, USA, 2017. Association for Computing Machinery.

[106] Hyo Jin Do. Intelligent interface for seeing the world through different lenses. In *Proceedings of the 22nd International Conference on Intelligent User Interfaces Companion*, IUI '17 Companion, page 217–220, New York, NY, USA, 2017. Association for Computing Machinery.

[107] David Gotz, Shun Sun, Nan Cao, Rita Kundu, and Anne-Marie Meyer. Adaptive contextualization methods for combating selection bias during high-dimensional visualization. *ACM Trans. Interact. Intell. Syst.*, 7(4), nov 2017.

[108] Simon Luo, Jianlong Zhou, Henry Been-Lirn Duh, and Fang Chen. Bvp feature signal analysis for intelligent user interface. In *Proceedings of the 2017 CHI Conference Extended Abstracts on Human Factors in Computing Systems*, CHI EA '17, page 1861–1868, New York, NY, USA, 2017. Association for Computing Machinery.

[109] Markus Schedl. Intelligent user interfaces for social music discovery and exploration of large-scale music repositories. In *Proceedings of the 2017 ACM Workshop on Theory-Informed User Modeling for Tailoring and Personalizing Interfaces*, HUMANIZE '17, page 7–11, New York, NY, USA, 2017. Association for Computing Machinery.

[110] Ivania Donoso-Guzmán. Epistaid: An interactive intelligent system for evidence-based health care. In *Proceedings of the 22nd International Conference on Intelligent User Interfaces Companion*, IUI '17 Companion, page 177–180, New York, NY, USA, 2017. Association for Computing Machinery.

[111] Shahram Eivazi, Wolfgang Fuhl, and Enkelejda Kasneci. Towards intelligent surgical microscope: Micro-surgeons' gaze and instrument tracking. In *Proceedings of the 22nd International Conference on Intelligent User Interfaces Companion*, IUI '17 Companion, page 69–72, New York, NY, USA, 2017. Association for Computing Machinery.

[112] Landy Rajaonarivo, Pierre De Loor, Eric Maisel, and Matthieu Courgeon. Using the enaction paradigm as a basis for database exploration to favor users sensemaking. page 224 – 227, 2017. Cited by: 0.

[113] Leszek Kaliciak, Hans Myrhaug, and Ayse Goker. Unified hybrid image retrieval system with continuous relevance feedback. volume 1, page 275 – 280, 2017.

[114] Hemilis Joyse Barbosa Rocha, Evandro de Barros Costa, Emanuele Silva, Natalia Caroline Lima, and Juliana Cavalcanti. A knowledge-based approach for personalised clothing recommendation for women. In *ICEIS*, 2017.

[115] Leszek Kaliciak, Hans I. Myrhaug, and Ayse Göker. Content-based image retrieval in augmented reality. In *ISAmI*, 2017.

[116] Sarang Shaikh, M. Ajmal Sawand, Najeed Ahmed Khan, and Farhan Badar Solangi. Comprehensive understanding of intelligent user interfaces. *International Journal of Advanced Computer Science and Applications*, 8, 2017.

[117] Christopher J. Hazard and Munindar P. Singh. Privacy risks in intelligent user interfaces. *IEEE Internet Computing*, 20(6):57–61, 2016.

[118] Agnieszka Landowska, Mariusz Szwoch, and Wioleta Szwoch. Methodology of Affective Intervention Design for Intelligent Systems. *Interacting with Computers*, 28(6):737–759, 10 2016.

[119] Jessica Rubart. Semantic adaptation of business information systems using human-centered business rule engines. In *2016 IEEE Tenth International Conference on Semantic Computing (ICSC)*, pages 187–193, 2016.

[120] Nan Yang and Amol D. Mali. Modifying keyboard layout to reduce finger-travel distance. In *2016 IEEE 28th International Conference on Tools with Artificial Intelligence (ICTAI)*, pages 165–168, 2016.

[121] Alexander Prange and Daniel Sonntag. Digital pi-rads: Smartphone sketches for instant knowledge acquisition in prostate cancer detection. In *2016 IEEE 29th International Symposium on Computer-Based Medical Systems (CBMS)*, pages 13–18, 2016.

[122] Ben Khayut, Lina Fabri, and Maya Avikhana. Modeling of computational systemic mind under uncertainty. *2016 IEEE 8th International Conference on Intelligent Systems (IS)*, pages 253–258, 2016.

[123] Suh-Yeon Dong, Bo-Kyeong Kim, and Soo-Young Lee. Eeg-based classification of implicit intention during self-relevant sentence reading. *IEEE Transactions on Cybernetics*, 46:2535–2542, 2016.

[124] Hang Guo. Deference and demeanor: Exploring interaction design for intelligent user interface. In *Proceedings of the 2016 ACM Conference Companion Publication on Designing Interactive Systems*, DIS '16 Companion, page 133–136, New York, NY, USA, 2016. Association for Computing Machinery.

[125] Pavel Andreevich Samsonov. Improving interactions with spatial context-aware services. In *Companion Publication of the 21st International Conference on Intelligent User Interfaces*, IUI '16 Companion, page 114–117, New York, NY, USA, 2016. Association for Computing Machinery.

[126] Maurício Sousa, João Vieira, Daniel Medeiros, Artur Arsenio, and Joaquim Jorge. Sleevear: Augmented reality for rehabilitation using realtime feedback. In *Proceedings of the 21st International Conference on Intelligent User Interfaces*, IUI '16, page 175–185, New York, NY, USA, 2016. Association for Computing Machinery.

[127] Jianlong Zhou, Syed Z. Arshad, Kun Yu, and Fang Chen. Correlation for user confidence in predictive decision making. In *Proceedings of the 28th Australian Conference on Computer-Human Interaction*, OzCHI '16, page 252–256, New York, NY, USA, 2016. Association for Computing Machinery.

[128] Ziyu Wang, Frank Hutter, Masrour Zoghi, David Matheson, and Nando De Freitas. Bayesian optimization in a billion dimensions via random embeddings. *J. Artif. Int. Res.*, 55(1):361–387, jan 2016.

[129] Mingkun Gao. Intelligent interface for organizing online social opinions on reddit. In *Companion Publication of the 21st International Conference on Intelligent User Interfaces*, IUI '16 Companion, page 134–137, New York, NY, USA, 2016. Association for Computing Machinery.

[130] Jong-Hyeok Lee, Kalyan Goswami, Byung-Gyu Kim, Seyoon Jeong, and Jin Soo Choi. Fast encoding algorithm for high-efficiency video coding (hevc) system based on spatio-temporal correlation. *Journal of Real-Time Image Processing*, 12(2):407–418, Aug 2016.

[131] Katrien Verbert, Denis Parra, and Peter Brusilovsky. Agents vs. users: Visual recommendation of research talks with multiple dimension of relevance. *ACM Trans. Interact. Intell. Syst.*, 6(2), jul 2016.

[132] Nina Runge, Pavel Samsonov, Donald Degraen, and Johannes Schöning. No more autobahn! scenic route generation using googles street view. In *Proceedings of the 21st International Conference on Intelligent User Interfaces*, IUI '16, page 147–151, New York, NY, USA, 2016. Association for Computing Machinery.

[133] S. Ravi Kumar, Md. Abdul Muqsit Khan, K. Chandra Sekharaiah, and Y. K. Sundara Krishna. A study on agent based ergonomic and intelligent user interface design in cloud computing. In *Proceedings of the Second International Conference on Information and Communication Technology for Competitive Strategies*, ICTCS '16, New York, NY, USA, 2016. Association for Computing Machinery.

[134] Mohammad Mehdi Moniri, Andreas Luxenburger, Winfried Schuffert, and Daniel Sonntag. Real-Time 3D Peripheral View Analysis. In Dirk Reiners, Daisuke Iwai, and Frank Steinicke, editors, *ICAT-EGVE 2016 - International Conference on Artificial Reality and Telexistence and Eurographics Symposium on Virtual Environments*. The Eurographics Association, 2016.

[135] George Azzopardi, Antonio Greco, and Mario Vento. Gender recognition from face images using a fusion of svm classifiers. In Aurélio Campilho and Fakhri Karray, editors, *Image Analysis and Recognition*, pages 533–538, Cham, 2016. Springer International Publishing.

[136] Michimasa Inaba, Kana Otsuka, and Kenichi Takahashi. Experimental investigation for a human relationship formation support agent using information presentation during conversation. In *Proceedings of the 8th International Conference on Agents and Artificial Intelligence*, ICAART 2016, page 87–94, Setubal, PRT, 2016. SCITEPRESS - Science and Technology Publications, Lda.

[137] Anastasios Karakostas, Ioulietta Lazarou, Georgios Meditskos, Thanos G. Stavropoulos, Ioannis Kompatsiaris, and Magda Tsolaki. Intelligent user interfaces to support diagnosis and assessment of people with dementia: An expert evaluation. In Silvia Serino, Aleksandar Matic, Dimitris Giakoumis, Guillaume Lopez, and Pietro Cipresso, editors, *Pervasive Computing Paradigms for Mental Health*, pages 196–206, Cham, 2016. Springer International Publishing.

[138] Alexander Prange and Daniel Sonntag. Easy deployment of spoken dialogue technology on smartwatches for mental healthcare. In *MindCare*, 2015.

[139] Rabail Tahir. Analyzing the intelligence in user interfaces. In *2015 SAI Intelligent Systems Conference (IntelliSys)*, pages 674–680, 2015.

[140] Katerina Kabassi and Maria Virvou. Combining decision-making theories with a cognitive theory for intelligent help: A comparison. *IEEE Transactions on Human-Machine Systems*, 45(2):176–186, 2015.

[141] Laurence Devillers, Sophie Rosset, Guillaume Dubuisson Duplessis, Mohamed A. Sehili, Lucile Béchade, Agnés Delaborde, Clement Gossart, Vincent Letard, Fan Yang, Yücel Yemez, Bekir B. Türker, Metin Sezgin, Kevin El Haddad, Stéphane Dupont, Daniel Luzzati, Yannick Esteve, Emer Gilmartin, and Nick Campbell. Multimodal data collection of human-robot humorous interactions in the joker project. In *2015 International Conference on Affective Computing and Intelligent Interaction (ACII)*, pages 348–354, 2015.

[142] Aleksandar Matic, Martin Pielot, and Nuria Oliver. Boredom-computer interaction: Boredom proneness and the use of smartphone. In *Proceedings of the 2015 ACM International Joint Conference on Pervasive and Ubiquitous Computing*, UbiComp '15, page 837–841, New York, NY, USA, 2015. Association for Computing Machinery.

[143] Sana Malik, Fan Du, Megan Monroe, Eberechukwu Onukwugha, Catherine Plaisant, and Ben Shneiderman. Cohort comparison of event sequences with balanced integration of visual analytics and statistics. In *Proceedings of the 20th International Conference on Intelligent User Interfaces*, IUI '15, page 38–49, New York, NY, USA, 2015. Association for Computing Machinery.

[144] Jianlong Zhou, Jinjun Sun, Fang Chen, Yang Wang, Ronnie Taib, Ahmad Khawaji, and Zhidong Li. Measurable decision making with gsr and pupillary analysis for intelligent user interface. *ACM Trans. Comput.-Hum. Interact.*, 21(6), jan 2015.

[145] Oswald Barral, Manuel J.A. Eugster, Tuukka Ruotsalo, Michiel M. Spapé, Ilkka Kosunen, Niklas Ravaja, Samuel Kaski, and Giulio Jacucci. Exploring peripheral physiology as a predictor of perceived relevance in information retrieval. In *Proceedings of the 20th International Conference on Intelligent User Interfaces*, IUI '15, page 389–399, New York, NY, USA, 2015. Association for Computing Machinery.

[146] Jared Alan Frank and Vikram Kapila. Path bending: Interactive human-robot interfaces with collision-free correction of user-drawn paths. In *Proceedings of the 20th International Conference on Intelligent User Interfaces*, IUI '15, page 186–190, New York, NY, USA, 2015. Association for Computing Machinery.

[147] M. Iftekhar Tanveer, Emy Lin, and Mohammed (Ehsan) Hoque. Rhema: A real-time in-situ intelligent interface to help people with public speaking. In *Proceedings of the 20th International Conference on Intelligent User Interfaces*, IUI '15, page 286–295, New York, NY, USA, 2015. Association for Computing Machinery.

[148] Nicolas Sabouret, Björn Schuller, Lucas Paletta, Erik Marchi, Hazaël Jones, and Atef Ben Youssef. Intelligent user interfaces in digital games for empowerment and inclusion. In *Proceedings of the 12th International Conference on Advances in Computer Entertainment Technology*, ACE '15, New York, NY, USA, 2015. Association for Computing Machinery.

[149] Lei Ren, Jin Cui, Ni Li, Qiong Wu, Cuixia Ma, Dongxing Teng, and Lin Zhang. Cloud-based intelligent user interface for cloud manufacturing: Model, technology, and application. *Journal of Manufacturing Science and Engineering-transactions of The Asme*, 137:040910, 2015.

[150] Anita Meier, Denys J. C. Matthies, Bodo Urban, and Reto Wettach. Exploring vibrotactile feedback on the body and foot for the purpose of pedestrian navigation. In *Proceedings of the 2nd International Workshop on Sensor-Based Activity Recognition and Interaction*, iWOAR '15, New York, NY, USA, 2015. Association for Computing Machinery.

[151] Evan M. Peck, Eleanor Easse, Nick Marshall, William Stratton, and L. Felipe Perrone. Flyloop: A micro framework for rapid development of physiological computing systems. In *Proceedings of the 7th ACM SIGCHI Symposium on Engineering Interactive Computing Systems*, EICS '15, page 152–157, New York, NY, USA, 2015. Association for Computing Machinery.

[152] James Schaffer, Prasanna Giridhar, Debra Jones, Tobias Höllerer, Tarek Abdelzaher, and John O'Donovan. Getting the message? a study of explanation interfaces for microblog data analysis. In *Proceedings of the 20th International Conference on Intelligent User Interfaces*, IUI '15, page 345–356, New York, NY, USA, 2015. Association for Computing Machinery.

[153] Lei Wang, Yixiong Liang, Wangyang Cai, and Beiji Zou. Failure detection and correction for appearance based facial tracking. *Chinese Journal of Electronics*, 24(1):20–25, 2015.

[154] Krzysztof Regulski, Gabriel Rojek, Dorota Wilk-Kołodziejczyk, Kluska-Nawarecka S., E. Nawarecki, and Grzegorz Dobrowolski. Intelligent interface for decision support system in metallurgical domain. *Computer Methods in Materials Science*, 15:71–77, 01 2015.

[155] Paul Taele and Tracy Hammond. Boponoto: An intelligent sketch education application for learning zhuyin phonetic script. pages 101–107, 09 2015.

[156] Antonio Jesus Fernandez-Garcia, Luis Iribarne, Antonio Corral, and James Z. Wang. Evolving mashup interfaces using a distributed machine learning and model transformation methodology. In Ioana Ciuciu, Hervé Panetto, Christophe Debruyne, Alexis Aubry, Peter Bollen, Rafael Valencia-García, Alok Mishra, Anna Fensel, and Fernando Ferri, editors, *On the Move to Meaningful Internet Systems: OTM 2015 Workshops*, pages 401–410, Cham, 2015. Springer International Publishing.

[157] Robbie T. Nakatsu. Enhancing the explanatory power of intelligent user interfaces with diagrams. In Fiona Fui-Hoon Nah and Chuan-Hoo Tan, editors, *HCI in Business*, pages 623–632, Cham, 2015. Springer International Publishing.

[158] Nesrine Mezhoudi, Iyad Khaddam, and Jean Vanderdonckt. Toward usable intelligent user interface. In Masaaki Kurosu, editor, *Human-Computer Interaction: Interaction Technologies*, pages 459–471, Cham, 2015. Springer International Publishing.

[159] Ali Danesh, Mukesh Kumar Saini, and Abdulmotaleb El Saddik. A proxemic multimedia interaction over the internet of things. In *MMM*, 2015.

[160] Zeynep Dogmus, Esra Erdem, and Volkan Patoglu. Rehabrobo-onto: Design, development and maintenance of a rehabilitation robotics ontology on the cloud. *Robotics and Computer-Integrated Manufacturing*, 33:100–109, 2015. Special Issue on Knowledge Driven Robotics and Manufacturing.

[161] Juan Costa-Dasilva, Alma Gómez-Rodríguez, Juan Carlos González Moreno, and David Valcárcel. A located and user personalized event's dissemination platform. *Journal of Intelligent and Fuzzy Systems*, 28:71–81, 01 2015.

[162] Steven Lawrence Fernandes and Josemin G. Bala. Low power affordable and efficient face detection in the presence of various noises and blurring effects on a single-board computer. In Suresh Chandra Satapathy, A. Govardhan, K. Srujan Raju, and J. K. Mandal, editors, *Emerging ICT for Bridging the Future - Proceedings of the 49th Annual Convention of the Computer Society of India (CSI) Volume 1*, pages 119–127, Cham, 2015. Springer International Publishing.

[163] Nilesh Chakraborty and Samrat Sohel Mondal. Color pass: An intelligent user interface to resist shoulder surfing attack. *Proceedings of the 2014 IEEE Students' Technology Symposium*, pages 13–18, 2014.

[164] Robert Ravnik, Borut Batagelj, Bojan Kverh, and Franc Solina. Dynamic anamorphosis as a special, computer-generated user interface. *Interacting with Computers*, 26(1):46–62, 2014.

[165] Andres Mejia-Figueroa and Reyes Juarez-Ramirez. Towards a user model for the design of adaptive interfaces for autistic users. In *2014 IEEE 38th International Computer Software and Applications Conference Workshops*, pages 264–269, 2014.

[166] Marcel Heupel, Mohamed Bourimi, and Dogan Kesdogan. The di.me trust approach for supporting collaborative scenarios. In *2014 25th International Workshop on Database and Expert Systems Applications*, pages 321–325, 2014.

[167] Rasam Bin Hossain, Mefta Sadat, and Hasan Mahmud. Recognition of human affection in smartphone perspective based on accelerometer and user's sitting position. In *2014 17th International Conference on Computer and Information Technology (ICCIT)*, pages 87–91, 2014.

[168] Freddy Lécué, Simone Tallevi-Diotallevi, Jer Hayes, Robert Tucker, Veli Bicer, Marco Luca Sbodio, and Pierpaolo Tommasi. Star-city: Semantic traffic analytics and reasoning for city. In *Proceedings of the 19th International Conference on Intelligent User Interfaces*, IUI '14, page 179–188, New York, NY, USA, 2014. Association for Computing Machinery.

[169] Adam Perer and Fei Wang. Frequence: Interactive mining and visualization of temporal frequent event sequences. In *Proceedings of the 19th International Conference on Intelligent User Interfaces*, IUI '14, page 153–162, New York, NY, USA, 2014. Association for Computing Machinery.

[170] Ingo R. Keck and Robert J. Ross. Exploring customer specific kpi selection strategies for an adaptive time critical user interface. In *Proceedings of the 19th International Conference on Intelligent User Interfaces*, IUI '14, page 341–346, New York, NY, USA, 2014. Association for Computing Machinery.

[171] Seiji Matsuyama, Takatomo Yamabe, Natsumi Takahashi, and Ryozo Kiyohara. Intelligent user interface of smartphones for on-vehicle information devices. *Procedia Computer Science*, 35:1635–1643, 2014. Knowledge-Based and Intelligent Information & Engineering Systems 18th Annual Conference, KES-2014 Gdynia, Poland, September 2014 Proceedings.

[172] H. Van Dyke Parunak, Marcus Huber, Randolph Jones, Michael Quist, and Jack Zaientz. Café: A group process to rationalize technologies in hybrid aamas systems. In Fabiano Dalpiaz, Jürgen Dix, and M. Birna van Riemsdijk, editors, *Engineering Multi-Agent Systems*, pages 227–245, Cham, 2014. Springer International Publishing.

[173] Lei Ren, Lin Zhang, Baocun Hou, Qiong Wu, and Dongxing Teng. Intelligent user interface in cloud manufacturing, Jun 2014.

[174] Daniel Sonntag and Daniel Porta. *Intelligent Semantic Mediation, Knowledge Acquisition and User Interaction*, pages 179–189. Springer International Publishing, Cham, 2014.

[175] David Leake, Ana Maguitman, and Thomas Reichherzer. Experience-based support for human-centered knowledge modeling. *Knowledge-Based Systems*, 68, 09 2014.

[176] Jacek Marciniak. Enhancing tagging systems by wordnet based ontologies. In Zygmunt Vetulani and Joseph Mariani, editors, *Human Language Technology Challenges for Computer Science and Linguistics*, pages 367–378, Cham, 2014. Springer International Publishing.

[177] Kyle Hipke, Michael Toomim, Rebecca Fiebrink, and James Fogarty. Beatbox: End-user interactive definition and training of recognizers for percussive vocalizations. In *Proceedings of the 2014 International Working Conference on Advanced Visual Interfaces*, AVI '14, page 121–124, New York, NY, USA, 2014. Association for Computing Machinery.

[178] Alexandros Papangelis, Georgios Galatas, Konstantinos Tsiakas, Alexandros Lioulemes, Dimitrios Zikos, and Fillia Makedon. A dialogue system for ensuring safe rehabilitation. In *Proceedings of the 8th International Conference on Universal Access in Human-Computer Interaction. Aging and Assistive Environments - Volume 8515*, page 349–358, Berlin, Heidelberg, 2014. Springer-Verlag.

[179] Reem Alnanih, Olga Ormandjieva, and Thiruvengadam Radhakrishnan. Empirical evaluation of intelligent mobile user interfaces in healthcare. In Marina Sokolova and Peter van Beek, editors, *Advances in Artificial Intelligence*, pages 23–34, Cham, 2014. Springer International Publishing.

[180] Xiaojun Bi, Tom Ouyang, and Shumin Zhai. Both complete and correct? multi-objective optimization of touchscreen keyboard. In *Proceedings of the SIGCHI Conference on Human Factors in Computing Systems*, CHI '14, page 2297–2306, New York, NY, USA, 2014. Association for Computing Machinery.

[181] Nicholas J. Bryan, Gautham J. Mysore, and Ge Wang. Isse: An interactive source separation editor. In *Proceedings of the SIGCHI Conference on Human Factors in Computing Systems*, CHI '14, page 257–266, New York, NY, USA, 2014. Association for Computing Machinery.

[182] Hugo Lopez-Tovar and John Dowell. A non-command interface for automatic document provision during meetings. In *Proceedings of the Companion Publication of the 19th International Conference on Intelligent User Interfaces*, IUI Companion '14, page 77–80, New York, NY, USA, 2014. Association for Computing Machinery.

[183] T.J. Gledhill, Eric Mercer, and Michael A. Goodrich. Modeling uass for role fusion and human machine interface optimization. In *2013 IEEE International Conference on Systems, Man, and Cybernetics*, pages 1929–1937, 2013.

[184] Dapeng Tao, Lianwen Jin, Weifeng Liu, and Xuelong Li. Hessian regularized support vector machines for mobile image annotation on the cloud. *IEEE Transactions on Multimedia*, 15(4):833–844, 2013.

[185] Zeynep Dogmus, Agis Papantoniou, Muhammed Kilinc, Sibel A. Yildirim, Esra Erdem, and Volkan Patoglu. Rehabilitation robotics ontology on the cloud. In *2013 IEEE 13th International Conference on Rehabilitation Robotics (ICORR)*, pages 1–6, 2013.

[186] Chiew Seng Sean Tan, Johannes Schöning, Kris Luyten, and Karin Coninx. Informing intelligent user interfaces by inferring affective states from body postures in ubiquitous computing environments. In *Proceedings of the 2013 International Conference on Intelligent User Interfaces*, IUI '13, page 235–246, New York, NY, USA, 2013. Association for Computing Machinery.

[187] Juho Kim, Haoqi Zhang, Paul André, Lydia B. Chilton, Wendy Mackay, Michel Beaudouin-Lafon, Robert C. Miller, and Steven P. Dow. Cobi: A community-informed conference scheduling tool. In *Proceedings of the 26th Annual ACM Symposium on User Interface Software and Technology*, UIST '13, page 173–182, New York, NY, USA, 2013. Association for Computing Machinery.

[188] Takushi Nishiyama, Munehiro Takimoto, and Yasushi Kambayashi. Human intervention for searching targets using mobile agents in a multi-robot environment. In *IIMSS*, 2013.

[189] Li Zhang. *Exploitation in Context-Sensitive Affect Sensing from Improvisational Interaction*, pages 12–23. Springer Berlin Heidelberg, Berlin, Heidelberg, 2012.

[190] Jochen Frey. Adapt – a dynamic approach for activity prediction and tracking for ambient intelligence. In *2013 9th International Conference on Intelligent Environments*, pages 254–257, 2013.

[191] Kaoru Sumi and Mizue Nagata. Characteristics of robots and virtual agents as a persuasive talker. In *Proceedings of the 7th International Conference on Universal Access in Human-Computer Interaction: User and Context Diversity - Volume 2*, UAHCI'13, page 414–423, Berlin, Heidelberg, 2013. Springer-Verlag.

[192] Marcel Heupel, Mohamed Bourimi, and Doğan Kesdoğan. Trust and privacy in the di.me userware. In Masaaki Kurosu, editor, *Human-Computer Interaction. Users and Contexts of Use*, pages 39–48, Berlin, Heidelberg, 2013. Springer Berlin Heidelberg.

[193] Kazuto Kurane, Munehiro Takimoto, and Yasushi Kambayashi. Design of an intelligent interface for the software mobile agents using augmented reality. In *ICAART*, 2013.

[194] Elizabeth M. Daly, Freddy Lecue, and Veli Bicer. Westland row why so slow? fusing social media and linked data sources for understanding real-time traffic conditions. In *Proceedings of the 2013 International Conference on Intelligent User Interfaces*, IUI '13, page 203–212, New York, NY, USA, 2013. Association for Computing Machinery.

[195] Isabelle Hupont, Sandra Baldassarri, and Eva Cerezo. Facial emotional classification: From a discrete perspective to a continuous emotional space. *Pattern Anal. Appl.*, 16(1):41–54, feb 2013.

[196] Stefan Ultes, Alexander Schmitt, and Wolfgang Minker. On quality ratings for spoken dialogue systems – experts vs. users. In *Proceedings of the 2013 Conference of the North American Chapter of the Association for Computational Linguistics: Human Language Technologies*, pages 569–578, Atlanta, Georgia, June 2013. Association for Computational Linguistics.

[197] A. A. Bashlykov. Visual representation of the condition of a complex technological controlled facility. *Scientific and Technical Information Processing*, 40(5):277–285, Dec 2013.

[198] Radu Orghidan, Mihaela Gordan, Marius Danciu, and Aurel Vlaicu. A prototype for the creation and interactive visualization of 3d human face models. In *Interdisciplinary Research in Engineering: Steps towards Breakthrough Innovation for Sustainable Development*, volume 8 of *Advanced Engineering Forum*, pages 45–54. Trans Tech Publications Ltd, 10 2013.

[199] Glenn Taylor, Richard Frederiksen, Jacob Crossman, Michael Quist, and Patrick Theisen. A multi-modal intelligent user interface for supervisory control of unmanned platforms. In *2012 International Conference on Collaboration Technologies and Systems (CTS)*, pages 117–124, 2012.

[200] Mohammed F. Alhamid, Mohamad Eid, and Abdulmotaleb El Saddik. A multi-modal intelligent system for biofeedback interactions. In *2012 IEEE International Symposium on Medical Measurements and Applications Proceedings*, pages 1–5, 2012.

[201] R. Jordon Crouser and Remco Chang. An affordance-based framework for human computation and human-computer collaboration. *IEEE Transactions on Visualization and Computer Graphics*, 18(12):2859–2868, 2012.

[202] Jeremy Ludwig and Eric Geiselman. Intelligent pairing assistant for air operation centers. In *Proceedings of the 2012 ACM International Conference on Intelligent User Interfaces*, IUI '12, page 241–244, New York, NY, USA, 2012. Association for Computing Machinery.

[203] Yen-Ling Kuo and Jane Yung-Jen Hsu. Planning for reasoning with multiple common sense knowledge bases. *ACM Trans. Interact. Intell. Syst.*, 2(3), sep 2012.

[204] Yuichi Murakami, Shingo Nakamura, and Shuji Hashimoto. An article kansei retrieval system combining recommendation function and interaction design. *Journal of Information Processing*, 20:548–558, 01 2012.

[205] Bernard Kamsu-Foguem, Germaine Tchuenté-Foguem, Laurent Allart, Youcef Zennir, Christian Vilhelm, Hossein Mehdaoui, Djamel Zitouni, Hervé Hubert, Mohamed Lemdani, and Pierre Ravaux. User-centered visual analysis using a hybrid reasoning architecture for intensive care units. *Decision Support Systems*, 54(1):496–509, 2012.

[206] Cláudia J. Abrão de Araújo and Flávio S. Corrêa da Silva. A system for governmental virtual institutions based on ontologies and interaction protocols. *Int. J. Distance Educ. Technol.*, 10(4):82–95, oct 2012.

[207] Nardjes Bouchemal and Ramdane Maamri. Sgp: Security by guaranty protocol for ambient intelligence based multi agent systems. In Sigeru Omatu, Juan F. De Paz Santana, Sara Rodríguez González, Jose M. Molina, Ana M. Bernardos, and Juan M. Corchado Rodríguez, editors, *Distributed Computing and Artificial Intelligence*, pages 289–296, Berlin, Heidelberg, 2012. Springer Berlin Heidelberg.

[208] Li Zhang. Contextual affect modeling and detection in interactive text-based dramatic improvisation. In Zhigeng Pan, Adrian David Cheok, Wolfgang Müller, Ido Iurgel, Paolo Petta, and Bodo Urban, editors, *Transactions on Edutainment X*, pages 36–52, Berlin, Heidelberg, 2013. Springer Berlin Heidelberg.

[209] Ruihai Dong, Kevin McCarthy, Michael O'Mahony, Markus Schaal, and Barry Smyth. Towards an intelligent reviewer's assistant: Recommending topics to help users to write better product reviews. In *Proceedings of the 2012 ACM International Conference on Intelligent User Interfaces*, IUI '12, page 159–168, New York, NY, USA, 2012. Association for Computing Machinery.

[210] Katharina Reinecke. Automatic adaptation of user interfaces to cultural preferences. *it - Information Technology*, 54:96–100, 04 2012.

[211] Martin Molina and Víctor Flores. Generating multimedia presentations that summarize the behavior of dynamic systems using a model-based approach. *Expert Syst. Appl.*, 39:2759–2770, 2012.

[212] Yolanda Gil, Varun Ratnakar, Timothy Chklovski, Paul Groth, and Denny Vrandecic. Capturing common knowledge about tasks: Intelligent assistance for to-do lists. *ACM Trans. Interact. Intell. Syst.*, 2(3), sep 2012.
